# From Mouse to Human: Evolutionary Genomics Analysis of Human Orthologs of Essential Genes

**DOI:** 10.1371/journal.pgen.1003484

**Published:** 2013-05-09

**Authors:** Benjamin Georgi, Benjamin F. Voight, Maja Bućan

**Affiliations:** 1 Department of Genetics, Perelman School of Medicine, University of Pennsylvania, Philadelphia, Pennsylvania, United States of America; 2 Department of Pharmacology, Perelman School of Medicine, University of Pennsylvania, Philadelphia, Pennsylvania, United States of America; The Wellcome Trust Centre for Human Genetics, University of Oxford, United Kingdom

## Abstract

Understanding the core set of genes that are necessary for basic developmental functions is one of the central goals in biology. Studies in model organisms identified a significant fraction of essential genes through the analysis of null-mutations that lead to lethality. Recent large-scale next-generation sequencing efforts have provided unprecedented data on genetic variation in human. However, evolutionary and genomic characteristics of human essential genes have never been directly studied on a genome-wide scale. Here we use detailed phenotypic resources available for the mouse and deep genomics sequencing data from human populations to characterize patterns of genetic variation and mutational burden in a set of 2,472 human orthologs of known essential genes in the mouse. Consistent with the action of strong, purifying selection, these genes exhibit comparatively reduced levels of sequence variation, skew in allele frequency towards more rare, and exhibit increased conservation across the primate and rodent lineages relative to the remainder of genes in the genome. In individual genomes we observed ∼12 rare mutations within essential genes predicted to be damaging. Consistent with the hypothesis that mutations in essential genes are risk factors for neurodevelopmental disease, we show that *de novo* variants in patients with Autism Spectrum Disorder are more likely to occur in this collection of genes. While incomplete, our set of human orthologs shows characteristics fully consistent with essential function in human and thus provides a resource to inform and facilitate interpretation of sequence data in studies of human disease.

## Author Summary

Essential genes are necessary for fundamental processes in an organism and lead to pre- or neonatal lethality when disrupted. In this work, we characterize 2,472 human orthologs of mouse essential genes in terms of their evolutionary and population genetics properties using data from recent deep sequencing initiatives in human populations. We find a signature of strong, purifying selection and a reduced load of sequence variants within the putative essential genes when compared to a control-group of non-essential genes. We also show a significant enrichment of variants within essential genes across a set of four recent studies of *de novo* variants in patients with Autism Spectrum Disorder. Our results establish the catalogue of putative essential genes as an important resource for analysis and interpretation of sequencing studies for human disease.

## Introduction

Next-generation sequencing (NGS) technologies are now routinely applied to evaluate the role of low-frequency and rare genetic variants in Mendelian and complex diseases [1]–[3]. Also, there is intense interest in utilizing large-scale NGS datasets to characterize the natural background and burden of sequence variation in the human genome. Historic estimates of the number of deleterious mutations per diploid human genome, based primarily on survival data from consanguineous marriages, vary significantly from 2–3 to 100 lethal equivalents, i.e. alleles or combinations of alleles that if made homozygous would be lethal [4]–[7]. The advent of large-scale NGS datasets allows for the first time to estimate the burden of variation in the human genome in a direct and unbiased manner. Recent studies leveraging NGS data to estimate the burden of damaging exonic missense variants report ∼400 such variants per human genome [7], [8]. With respect to loss-of-function (LoF) variants, a study of 185 human genome sequences finds a load of ∼100 high-confidence LoF variants per genome [9]. A recent study of autosomal recessive disease variants in a genetic isolate finds surprisingly high carrier frequencies for many of these variants [8]. Finally, a study of the evolutionary origins of human protein coding variants reports that 86% of putative deleterious variants are of very recent origin (5,000–10,000 years) [10].

A major challenge in analyzing NGS data for clinical applications is the identification of mutations likely implicated in disease among the hundreds of thousands of harmless variants, and only a few, modestly powered strategies have been described [2], [11], [12]. One model underlying most of these approaches is that variants disrupting gene function are more likely to have fitness consequences and thus, are more likely pathogenic. A complementary approach to these methods would instead infer sets of genes that are evolutionarily constrained in human populations directly, based on polymorphism data. If such gene sets could be credibly identified, this information could greatly benefit the interpretation, prioritization, or even association testing of genetic variation identified from sequencing studies for human disease.

One approach to identify a constrained set of genes comes from studies of essential genes in model organisms. Homozygous loss-of-function mutations in essential genes cause lethality during embryogenesis or shortly after birth. A large number of genes causing embryonic lethality have been identified by forward and reverse genetics experiments [13]. In the mouse, systematic analysis of targeted loss-of-function mutants uncovered almost 3,000 genes that are demonstrably essential for viable development, as introduction of homozygous loss-of function mutations cause lethality either during embryogenesis or before weaning [14]. Importantly, phenotypic analyses of heterozygous alleles of a set of 139 non-viable mutant mouse strains demonstrated at least one phenotype in 70% of the lines [15].

In humans, the role of essential genes is typically discussed in the context of association with disease. It has been shown that human disease genes which are also lethal in the mouse tend to be highly connected in protein-protein interaction networks, and more likely to demonstrate a dominant mode of inheritance than other human disease genes [16]. Similarly, a recent study focusing on rare genetic diseases reported an enrichment of essential causal genes among these so-called orphan diseases [17].

In this study, by taking advantage of available sequence data in humans from large-scale sequencing studies [2], [18], we aim to address two basic questions: are genes identified as ‘essential’ in the mouse also evolutionarily conserved in humans, and second, how is this reflected in their mutational burden and impact on human disease? Our results show strong and consistent signatures of purifying selection within the set of essential genes, including increased sequence conservation, reduced number of exonic missense variants and an overall shift in allele frequency towards rare alleles. Leveraging these results, we then show that *de novo* mutations in Autism Spectrum Disorder (ASD) cases are significantly enriched in this gene set in data from recent papers related to ASD. Our strategy highlights the importance of model system biology and gene set classification for disease studies in humans.

## Results

**Figure 1:**
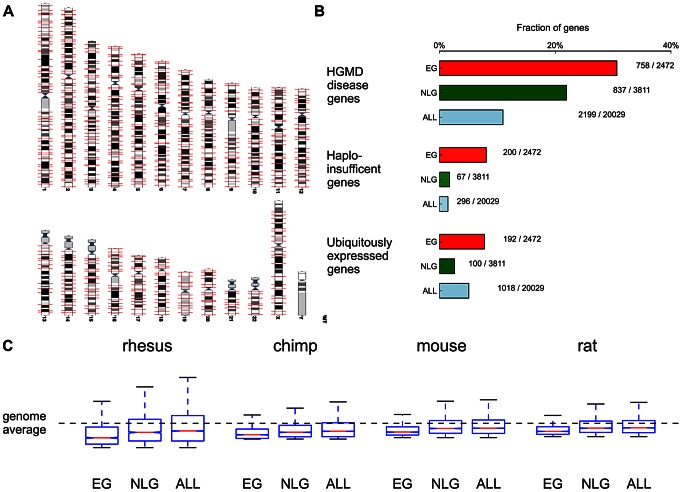
Functional and evolutionary characteristics of essential genes. A) Distribution of the 2472 essential genes (EG) across the genome (obtained from www.ensembl.org). B) Essential genes are significantly enriched in HGMD disease genes (*P* = 1.73×10^−14^), haploinsufficient genes [21] (*P* = 1.75×10^−33^) and ubiquitously expressed genes (*P* = 9.23×10^−21^) when compared to NLG. C) Comparison of non-synonymous to synonymous substitution rates between human and rhesus, chimp, mouse and rat in EG, NLG and ALL. Plotted substitution rates are normalized to Z-scores relative to the genome average.

By surveying genotype-phenotype associations in the Mouse Genome Informatics database (MGI) [13] we identified 2,485 mouse genes associated with 46 phenotypic categories (pre-, peri- and postnatal lethality). From the collection of 20,029 protein coding genes (ALL) retrieved from the GENCODE database [19], we extracted the set of 2,472 one-to-one human orthologs of these essential mouse genes (EG, Figure 1A, Methods) and the non-overlapping set of 3,811 human orthologs of genes with known non-lethal phenotype (NLG) in homozygous mouse mutants (Table S6). If a null-mutant for a specific gene had both lethal and non-lethal phenotypes annotated in the database, it was assigned to the EG set. We noted several features that characterize the set of EG, namely (a) an enrichment of functional categories related to gene expression, cell growth, cell death, cell proliferation and cancer based on Ingenuity Pathway analysis (Figure S1); (b) an enrichment of EG relative to NLG in disease genes based on variants cataloged in the Human Genome Mutational Database [20] (Fisher's Exact *P* = 1.73×10^−14^, OR = 1.57, 95% CI 1.39–1.77, Table S2A); (c) an enrichment of EG relative to NLG for haploinsufficient genes [21] (Fisher's Exact *P* = 1.75×10^−33^, OR = 4.91, 95% CI 3.69–6.62, Figure 1B, Table S2B); (d) an enrichment of EG relative to NLG within the top 10% of ubiquitously expressed genes [22] (Fisher's Exact *P* = 9.23×10^−21^, OR = 3.12, 95% CI 2.43–4.04, Table S2C), with a subset of essential genes showing remarkable tissue-specific expression both in the mouse and in humans (Figures S2, S3, S4) and (e) an increase in the number of alternative transcripts (Wilcoxon *P* = 2.19×10^−23^) in EG relative to NLG (see also Text S1). These features are consistent with the hypothesis that experimentally validated essential genes in the mouse are functionally important in humans.

Due to the central role of EG in a rodent (mouse), we hypothesized that these same genes should be subject to heightened evolutionary constraint across the primate lineage. First, when comparing non-synonymous to synonymous substitution rates between human and two primate (rhesus, chimp) and two rodent species (mouse and rat), we consistently observed significantly lower dN/dS ratios, indicating stronger evolutionary constraint, in EG compared to NLG (e.g. Wilcoxon *P* = 5.15×10^−34^ for rhesus monkey, Figure 1C, Table S1). Second, based on PhlyoP [23] nucleotide conservation scores across nine primate species, we observed significantly more constraints in EG when compared to NLG for both exonic regions (Wilcoxon *P* = 1.28×10^−75^, Figure S5) as well as promoter regions (Wilcoxon *P* = 7.93×10^−6^, Figure S6).

It has been reported that brain expressed genes are subject to increased purifying selection [24]–[26]. We asked a) whether genes over-expressed in the adult brain are enriched among EG and b) whether any enrichment in brain over-expressed genes within EG may account for the increased level of purifying selection observed in the EG set. To address this question we retrieved a list of 2,249 human brain over-expressed genes identified in a study of 79 human tissues and cell lines [27] from the Gene Expression Atlas (http://www.ebi.ac.uk/gxa/). A threshold of *P*< = 1e-4 was applied to identify genes with highly significant over-expression in the brain. We then selected genes that are over-expressed in the brain that are EG (399/2,472) and NLG (588/3,811). There was no enrichment of brain over-expressed genes in EG versus NLG (Fisher's Exact *P* = 0.46, OR = 1.05, 95% CI 0.92–1.21), nor did the stratification change the relatively higher conservation of exons (Wilcoxon *P* = 4.84×10^−7^) or reduced dN/dS ratio (Wilcoxon *P* = 2.24×10^−6^ for the mouse-human comparison) in essential genes. Taken together these results show that the signature of strong purifying selection in essential genes remains when controlling for over-expression in the brain. However, when comparing dN/dS ratios across all four species (rhesus, chimp, mouse and rat) for 399 essential genes with brain over-expression and the remaining 2,073 genes in EG, we observed stronger selection on the EG over-expressed in brain (e.g. Wilcoxon *P* = 1.14×10^−18^ for the mouse-human comparison). This suggests that within the set of essential genes, brain over-expressed genes form a subset with particularly strong purifying selection.

**Figure 2:**
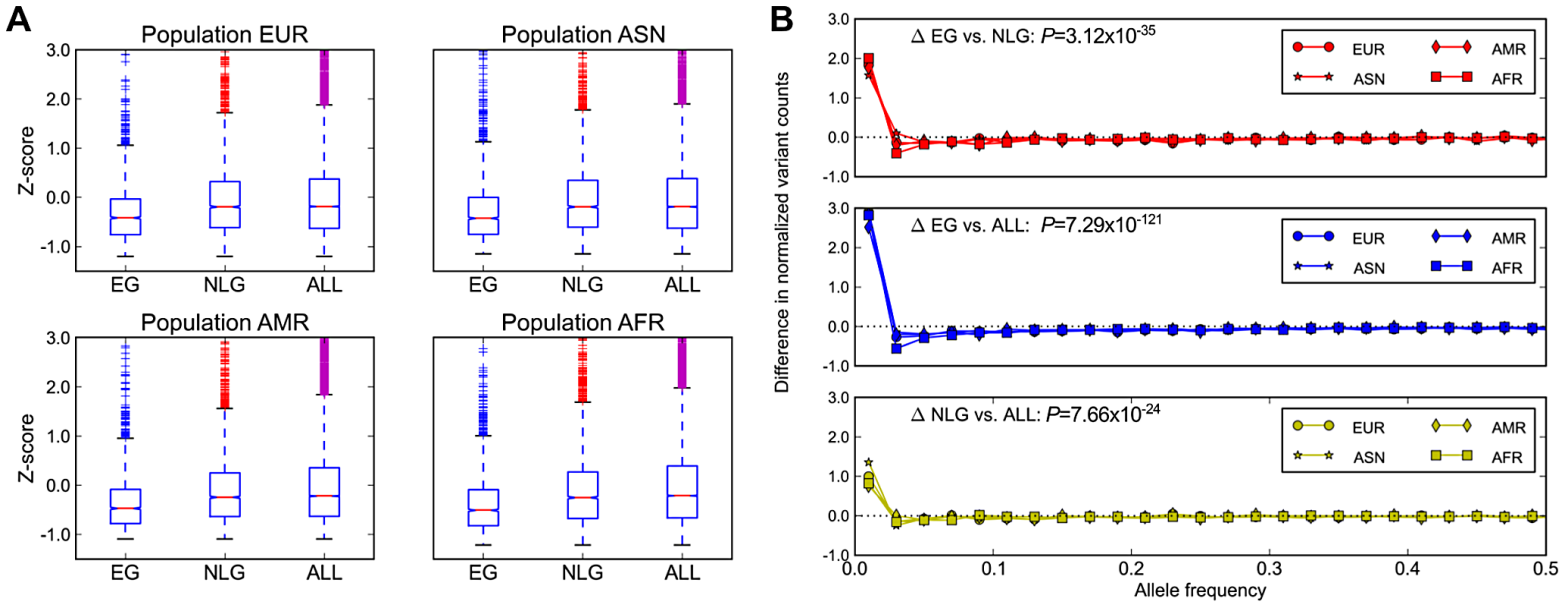
Population genetics properties of essential genes. A) Average numbers of exonic missense variants in EG, NLG and ALL. The plotted Z-score is normalized relative to the genome average. The plotted range is truncated to visualize differences between gene sets, with a full log-transformed plot available in Figure S7. B) Differences in the allele frequency distributions in four continental populations of the 1000G data for EG, NLG and ALL. A data point above the zero line corresponds to a relative excess of variants of a given allele frequency. It can be seen that the essential genes contain significantly more rare variants than either NLG or ALL. The reported p-values are with respect to all 1000 Genome samples combined.

In addition to evolutionary constraint across species, we hypothesized that genes identified as essential in the mouse should also be subject to significant background selection in recent human history. This pressure would be expected to leave a signature of (a) a reduction in overall polymorphism levels, particularly in the levels of missense and loss-of-function mutations, and (b) a skewing of the allele frequency distribution towards increasingly rare variants in EG relative to NLG. Using data from the 1000 Genomes Project [18] Phase 1 release, and after controlling for the total exon length in each gene, we observed a significant reduction in the level of exonic single nucleotide polymorphisms (SNP) in EG relative to either NLG or ALL (Wilcoxon Test *P* = 1.08×10^−59^, Figure 2A) as well as a shift in the distribution of allele frequencies towards rare variants (Wilcoxon Test *P* = 3.12×10^−35^, Figure 2B, Figure S8). Both of these results hold even after stratifying by continental population group (Asian, African, American or European) or when considering individual subpopulations (Table S3, Table S4). To ensure that this observed constraint is not simply a result of the sequencing technology used to produce the data or the number of individuals characterized, we also examined re-sequencing data reported recently for ∼200 drug-target genes in 14,002 individuals [28]. After adjusting for total exon length, we confirmed a significant reduction in the level of polymorphisms among 55 EG compared to 115 NLG in this set of genes (Wilcoxon Test *P* = 9.78×10^−7^).

**Figure 3:**
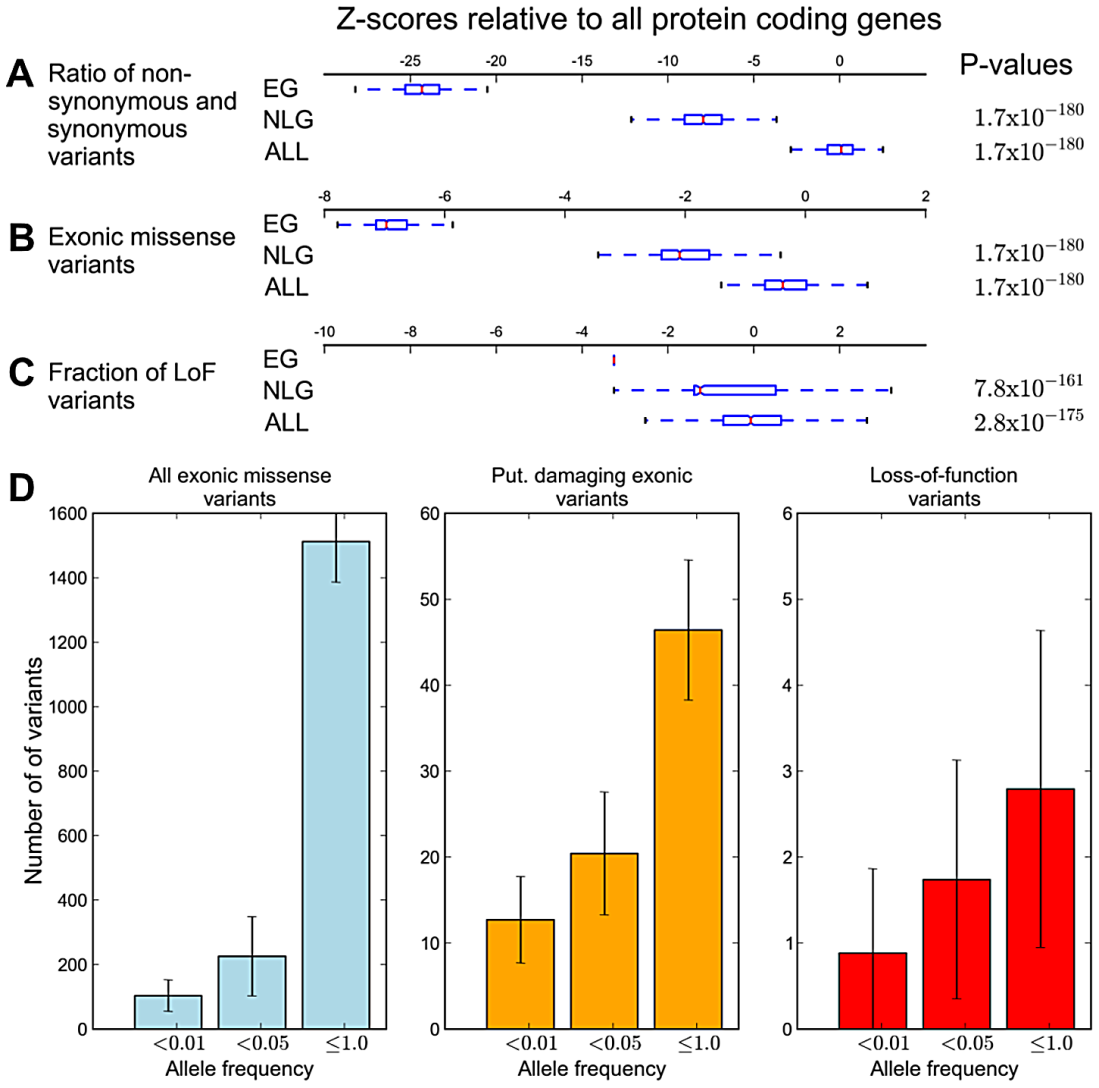
Analysis of individual mutational load in essential genes. The boxes span the lower and upper quartile with the median indicated by a red bar; whiskers extend to data points less than 1.5 times the interquartile range. Values are transformed to Z-scores relative to the genome average of all protein coding genes. The P-values given are for the comparison of EG versus NLG (top) and EG versus ALL (bottom). A) Ratio of non-synonymous to synonymous exonic variants. B) Gene-length corrected average number of exonic missense variants. C) Fraction of loss-of-function variants among all exonic missense variants. D) Estimates of mutational load in essential genes in each human genome at different allele frequencies. The plots show all exonic missense variants (blue), putative damaging exonic variants (orange) and loss-of-function variants (red). Error bars depict the standard deviation.

Under the model that a subset of mutations in essential genes are subject to purifying selection at a population level, we hypothesized that across the set of essential genes, individual genomes should also exhibit reduced mutational load. When comparing the mutational load in essential genes for each sample in the 1000 Genomes Phase 1 data, we observed a significant reduction in the ratio of non-synonymous to synonymous substitution within EG compared to NLG (Wilcoxon *P* = 1.66×10^−180^, Figure 3A), as well as an overall reduction in the number of missense variants (Wilcoxon *P* = 1.66×10^−180^, Figure 3B). The observed constraint on polymorphisms in essential genes suggests a higher rate of deleterious mutations removed from the population by background selection, and thus, a lower incidence of severe effect variants in each individual genome. To test this hypothesis, we investigated the difference in the relative abundance of loss-of-function (or LoF variants), i.e. variants that introduce or disrupt a stop codon (nonsense or read-through) or splicing sites. The comparison of the fraction of LoF variants among all exonic non-synonymous SNPs within each group showed a significantly lower fraction of LoF events in EG compared to NLG (*P* = 6.38×10^−161^ paired Wilcoxon test, Figure 3C). In fact, only 11% (122) of samples did have a LoF event for any gene in EG, compared to 96% (1045) of samples for the NLG set. Similarly, we observed a striking, almost 5-fold increase in the ratio of heterozygous to homozygous LoF variants within EG (22.25) to NLG (4.53) (Wilxocon P = 3.71×10^−111^). These findings are consistent with data from a recent study of LoF variants in 185 human genomes [9]. From the 1,102 genes reported to be hit by high confidence LoF variants, 190 belonged to either the EG or NLG classes. We observed a depletion of high confidence LoF variants within EG versus NLG (Fisher's exact test *P* = 0.012, OR = 0.67, 95% CI 0.48–0.92, Table S2D). Thus, there is not only an overall reduction in the number of exonic missense variants in EG, but the variants that are present also tend to be less severe. To provide additional technical validation of our observations, we repeated the analysis using the whole-genome sequence of 54 HapMap individuals made available by Complete Genomics ([29], http://www.completegenomics.com/public-data/69-Genomes/) and observed comparable results (see Text S1, Figures S9 and S10, Tables S9 and S10).

The 1000 Genomes data also provided an opportunity to quantify the mutational burden within essential genes. To accomplish this, we calculated average counts per individual of (a) missense variants, (b) putatively damaging variants identified by a consensus of PolyPhen2 [12] and SIFT [11] and (c) high-confidence LoF variants (Tables S7 and S8). In order to put our estimate of mutational burden within essential genes in the context of previous predictions, we first calculated estimates for all protein-coding genes. Within ALL, we observe an average of 449.82 (SD 82.02) putative damaging exonic variants. Prior estimates place this number at ∼400 variants per human genome [7], [8]. The average number of putative LoF events is 59.03 (SD 11.48) somewhat lower than a recent report of ∼100 high-confidence LoF variants per human genome. The same study showed that true LoF events are highly enriched for rare alleles [9]. Thus we highlight rare (<1%) LoF variants and observe an average of 12.84 (SD 5.71) spread over ∼13 genes. The overall burden in EG consists of 1,137 (SD 216.11) exonic missense variants in ∼665 genes. This includes ∼40.34 (SD 9.48) putatively damaging variants and ∼2.79 (SD 1.84) LoF events (Figure S11). For rare variants (<1%), we found ∼89.57 (SD 42.88) exonic missense variants, including ∼11.61 (SD 4.88) putative damaging variants and ∼0.88 (SD 0.98) LoF events, giving a mutational burden estimate in essential genes of ∼12 per individual.

**Figure 4:**
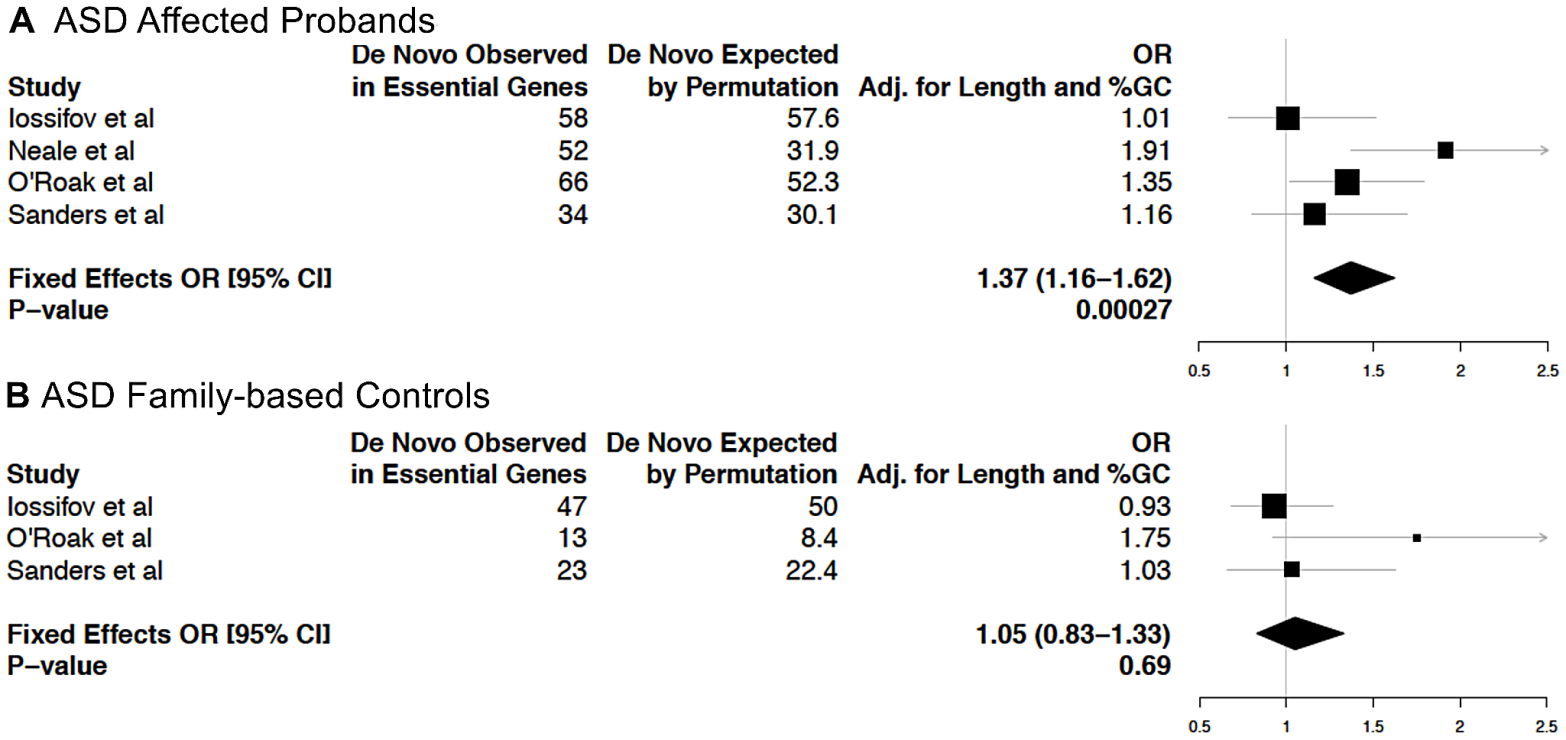
Analysis of enrichment of essential genes among genes with ***de novo*** **mutations in ASD families from four recent studies.** Gene-length and GC content adjusted odds ratios (OR) and P-values for enrichment in EG in either (A) ASD affected probands or (B) family-based controls (i.e. unaffected siblings) are shown.

Recently, four studies investigated the role of *de novo* mutations in disease risk in probands of families segregating ASD [1], [30]–[32]. Many genes predisposing an individual to ASD have a crucial role during neuronal development and in the formation of neuronal circuits in the early postnatal period [33]. For a recently reported set of 112 ASD candidate genes selected based on published reports [31], we observed an enrichment of EG compared to NLG (Fisher's exact test *P* = 0.001, OR = 2.08, 95% CI 1.31–3.3, Table S2E). Thus, hypothesizing that mutations in essential genes are more likely to predispose to a neurodevelopmental disorder such as ASD, we computed the rates of *de novo* mutations from these four studies in affected probands relative to family-based controls. Considering mutations across coding transcripts and splice sites, and using a gene-based permutation procedure matching total exon lengths and %GC content, we observed an enrichment of *de novo* mutations in affected individuals at essential genes (*P* = 2.7×10^−4^, adjusted OR: 1.37, 95% CI: 1.16–1.62, Figure 4A), but not among the NLG gene set (*P* = 0.91, adjusted OR: 0.99, 95% CI: 0.83–1.18) nor in family-based controls (*P* = 0.69, adjusted OR: 1.05, 95% CI: 0.83–1.33, Figure 4B). A more conservative approach that removed synonymous mutations still demonstrates a nominal enrichment in affected individuals (*P* = 0.038, adjusted OR: 1.23, 95% CI: 1.01–1.50), but not in the NLG gene set (*P* = 0.88, adjusted OR: 1.02, 95% CI: 0.83–1.24) nor in controls (*P* = 0.25, adjusted OR: 1.17, 95% CI: 0.89–1.53) for essential genes. We also performed permutation experiments analogous to the above, focusing solely on *de novo* mutations in the subset of essential genes with overexpression in the brain. None of these comparisons were significantly enriched (*P*>0.05). This is not altogether unexpected because the number of *de novo* mutations examined in this subset was 10–20% of the total number of events, presumably resulting in insufficient power for the test (data not shown). Among the 259 essential genes with *de novo* events, 179 genes are hit by events exclusively in ASD cases (Table S11). Analysis of protein-protein interactions between these essential genes using DAPPLE [34] revealed enrichment in connectivity (*P* = 0.0019 based on 1,000 permutations, Figure S12A). Also, DAPPLE analysis of the 399 brain-overexpressed essential genes showed enrichment in connectivity (*P* = 0.0009 based on 1,000 permutations, Figure S12B). Encouragingly, of the two most significantly connected essential genes with *de novo* variants in ASD (*P*<0.002: *CTNNB1* and *HDAC1*), links between ASD and interaction networks including *CTNNB1* have been previously highlighted [32]. A manual survey of genotype-phenotype associations of the 179 genes in the MGI database showed that 32/179 essential genes have reported heterozygote phenotypes, including behavioral and neurological anomalies (e.g *ANK2*, *AP3DI*, *CACNA2D2*, *CD717*, *CTNNBI*, *DGCR8*, *DYRKIA*, *GNAS*, *MCOLN3*, *NR4A2*, *RIC8A*, *SLC17A6* and *SMARCCI*). Nine out of the 179 essential genes are also ASD candidates (*PTEN*, *FOXP1*, *CHD7*, *MEF2C*, *STXBP1*, *TSC2*, *CASK*, *GRIN2B* and *SCN1A*). These genes, as well as others from the essential gene network, can be viewed as priority targets for further work to deduce the biological rationale and functional meaning of the observed enrichment.

## Discussion

We compared a broad range of evolutionary and population genetic characteristics of 2,472 putative essential human genes with a set of non-essential genes. This analysis shows a consistent and strong footprint of purifying selection in the essential genes: higher sequence conservation (both exons and promoter), reduced non-synonymous/synonymous substitution rates across the rodent and primate lineage, reduced load of non-synonymous sequence variants and a shift towards rarer allele frequencies. All of our analyses clearly and consistently support the functional significance of the EG set in humans.

Our findings are based on a catalog of 2,472 essential genes identified by phenotypic profiling of primarily loss-of-function mutants reported in the Mouse Genome Informatics database [13]. Systematic and comprehensive phenotyping of a large collection of recently generated mutant lines will soon be available [14], [35]. Based on an estimate from a pilot study, roughly 30% of genes in the mouse genome may be necessary for survival to adulthood [15]. Therefore, our current catalog of over 2,400 human orthologs of essential genes in the mouse is far from complete. Another limitation of our approach is the uncertainty regarding the extent of conservation of null-phenotypes of orthologs across species. In a study of 120 human disease genes, considered essential based on clinical features of death before puberty, more than 20% of mouse orthologs have reported non-lethal phenotypes [36]. However, cross-species comparisons are complicated by the inability to unequivocally assess essentiality of specific alleles in humans. Moreover, in contrast to experimentally confirmed null-mutations in a model organism [16], in humans it is often difficult to decipher the nature of mutations and distinguish single-gene and polygenic effects. For these reasons, conservation of lethal phenotypes from mouse to human has, to the best of our knowledge, never been systematically studied. A similarity in the expression pattern may, to some extend, suggest similar function. It has been shown that 79% of 995 genes important for neural functions have highly conserved expression patterns in human and mouse [37]. Similarly, a study of 799 human and mouse orthologes in 16 tissues showed that half of the orthologues were expressed with correlations of 0.6 or better [38]. Finally, a comparative study of region-specific gene expression in human and mouse brain regions found that corresponding brain regions had similar expression profiles [39]. For the essential genes we confirmed strong concordance in the direction of differential expression (up- or down regulation) in mouse and human (see Text S1).

The estimate of a total load of ∼12 predicted damaging exonic variants per individual in 2,472 human orthologs of essential genes represent the first attempt to directly estimate the individual mutational burden in putative human essential genes at a molecular level. The assessment of the overall burden of ∼400 damaging exonic variants is in line with recent estimates [7], . The number of ∼60 high-confidence LoF events per sample in all protein-coding genes somewhat deviates from the previously reported ∼100. However, this number was based on data from a single, high-coverage genome sequence whereas our estimate takes into account all 1092 samples of the 100 Genomes phase 1 release [18]. Since our list of essential genes is incomplete, and because the functional impact of individual variants is based on predictive evidence, quantifying the exact estimate of lethal equivalents in each individual will require a combination of sequencing and experimental studies.

To be explored further is the difference in the burden of homozygous versus heterozygous amino-acid changing substitutions in human orthologs of essential genes in a model organism. Although, as expected, we observed significantly less putative damaging variants in homozygous states in 2,472 essential genes, our ability to interpret these differences is limited for several reasons. First, detected homozygous mutations in essential genes may not effect gene or protein function due to erroneous labeling as “damaging” by Polyphen2, SIFT or similar algorithms [40]. Also, as evidenced in model organisms ([41], [42] and references therein), heterozygous mutations in essential genes may be also lethal (haplolethal) or under negative selection. Finally, a combination of non-lethal mutations in a combination of essential or non-essential interacting genes may lead to the lethality (synthetic lethality) [43]. On a related note, our systematic analysis of genes associated with lethality in the MGI database identified ∼400 mouse genes with viable knockout mutations but reported lethality of combinations of these mutations with knockout alleles for other genes (data not shown).

The application of our set of essential genes to the ASD *de novo* datasets [1], [30]–[32] can be seen as a proof-of-concept how leveraging prior knowledge on gene essentiality can improve and facilitate the interpretation of results from clinical sequencing studies. The observed significant enrichment of ASD *de novo* variants in essential genes in turn means that these variants should be prioritized in downstream analyses and follow-up experiments. With sufficiently large data sets in the future, it should be possible to construct “prioritization” tables that document the enrichment of different classes of mutations – loss-of-function, missense, etc. – in the context of gene sets – Essential versus not-Essential. Such an empirical table of prior scores would be a highly useful and quantitative approach for researchers in facilitating design of follow-up studies. Recent genetic studies of ASD revealed that hundreds of genes could be involved in ASD pathophysiology [44], [45]. Many of the genes implicated in ASD have a role in neuronal development, synapse formation and synapse function. Our finding that essential genes are overrepresented among genes with *de novo* mutations in ASD may influence our thinking about the timing of molecular anomalies leading to behavioral consequences. Instead of focusing exclusively on neurodevelopmental processes during the early postnatal period, our findings suggest a possible role for both genetic and environmental influences during embryonic or prenatal development. Our results also suggest that heterozygosity at one or (more likely) multiple essential genes should be considered as disease-causing. Although homozygous null-mutations in these genes lead to embryo death (in the mouse and presumably in humans), a haploinsufficiency for a null-allele or a spectrum of alleles other than null may contribute to behavioral anomalies. Finally, clinical comorbidities and phenotypic heterogeneity, which represent a hallmark for ASD, may be explained by a combined, possibly pleiotropic effect of rare heterozygote mutations in several genes with a key role in embryonic or prenatal development. However, it is clear that the observed overrepresentation of essential genes among genes with *de novo* mutations in ASD represents only a preliminary finding, which highlights the importance of further analysis of genetic variation, especially *de novo* versus inherited mutations, in essential genes in ASD subjects, their relatives and control subjects.

It is important to note that the samples in the 1000 Genomes Project were collected without phenotypic information or, if such information was available, it was not shared with the 1000 Genomes team. As such, each of the putatively damaging and loss-of-function variants found within these samples is less likely to be directly causal for a disease. However, given the reduced number of non-synonymous events within essential genes and the stronger selection acting on these genes, systematic analysis of these, often rare variants in individuals with known medical history, may reveal new disease risk or protective alleles.

The results of this study establish a set of essential genes as being of both biological as well as practical importance for facilitating the interpretation of next-generation sequencing studies. By making use of *a priori* knowledge of key biological properties of genes, the ability to prioritize and interpret the occurrence of specific variants within a gene is improved. However, it is clear that our list represents only the first step towards a catalogue of essential human genes and considerable additional work will be required to refine the functional annotation of specific genes.

## Methods

### Essential genes

We extracted genes with known lethal phenotype from the Mouse Genome Database (MGD) [13] on the Mouse Genome Informatics (MGI) website (ftp://ftp.informatics.jax.org/pub/reports/MGI_PhenoGenoMP.) Genes annotated with any of 46 prenatal, perinatal or postnatal lethal phenotypes (Table S5) and unambiguous chromosomal positions were considered to be essential. We identified 2,485 essential mouse genes, 2,472 of which could be mapped to a direct human ortholog using information in the MGI database. Orthology assignments from the MGI database were checked against data in Ensembl. Lethal phenotypes for the identified essential genes were manually confirmed in the MGI phenotype summaries. As a negative set and basis of comparison, we also identified 3,863 mouse genes where non-lethal phenotypes have been reported in the MGI database. The non-essential mouse genes could be mapped to 3,811 human orthologs. Thus, mining of the MGI database resulted in 2,472 essential genes (EG) and 3,811 genes with non-lethal phenotypes (NLG) in human based on experimental evidence in mouse. Phenotypes that were observed only in a heterozygote state were disregarded. As an additional control we used the full set of 20,029 protein coding genes in the human genome (ALL) retrieved from the GENCODE 13 database [19]. Annotations for all genes including essentiality status are given in Table S6. A comparison of the length of coding sequence in EG, NLG and ALL showed that on average genes in EG are significantly longer than the genomic average (EG versus ALL, *P* = 1.73×10^−92^, Wilcoxon test, Figure S13). This underlines the importance of accounting for gene size effect in all analyses. As GC content is positively correlated with mutation rate, we next retrieved the average GC content of all genes from Ensembl (www.ensembl.org/biomart/martview). While we find a slightly lowered GC content in EG compared to NLG (*P* = 0.00083, Wilcoxon test), there is no difference when compared to the genomic average (*P* = 0.38, Wilcoxon test, Figure S14).

### 1000 Genome Phase 1 variant data

Variant calls from the 1000 Genomes project [18] Phase 1 release v3 were obtained from the EBI ftp server (ftp://ftp.1000genomes.ebi.ac.uk/vol1/ftp/release/20110521/). The Phase 1 data encompasses 1092 samples from four subpopulations: African (AFR), Asian (ASN), American-admixture (AMR) and European (EUR) with all genotypes phased and imputed. This release does not include genotype data for chromosome Y and genes on that chromosome were thus excluded from the analysis of the dataset. Variants were annotated using the SNPEff (v2.1b) software (http://snpeff.sourceforge.net/). The annotated variants were filtered to retrieve exonic SNP variants in protein coding genes. Exonic missense variants were identified by variant types “SPLICE_SITE_ACCEPTOR”, “SPLICE_SITE_DONOR”, “STOP_GAINED”, “NON_SYNONYMOUS_CODING”, “STOP_LOST”, “START_LOST” and “START_GAINED”. Polyphen2 [12] and SIFT [11] predictions for all non-synonymous variants were retrieved from the dbNSFP v2.0b3 database [46]. Only variants with a consensus of both algorithms were considered putatively damaging. A curated list of high-confidence loss-of-function variants was obtained from the 1000 Genomes server (http://ftp.1000genomes.ebi.ac.uk/vol1/ftp/phase1/analysis_results/functional_annotation/annotated_vcfs/ALL.wgs.integrated_phase1_release_v3_Loss_of_Function_20120626.20101123.xls). Allele frequencies for all variants were computed using the vcftools (v0.1.9.0) software (http://vcftools.sourceforge.net) –freq option. To test for systematic bias of genotype call quality between the EG, NLG and ALL gene sets, we compared gene-wise averages of the average imputation genotype posterior probabilities (“AVGPOST” in the vcf files) over all samples. We did not observe any significant difference of genotype quality scores between EG and either NLG (Wilcoxon test, P = 0.106) or ALL (Wilcoxon test, P = 0.444).

### Human Genome Mutation Database (HGMD)

A list of 52,254 disease linked variants were obtained from the HGMD website (http://www.hgmd.org/). Annotated disease causing variants (DM) were mapped to 2220 different genes. These genes were then used to test for enrichment of essential genes by 2×2 contingency table analysis using Fisher's exact test.

### Gene expression data

To address gene expression patterns of essential genes, we obtained a list of 13,629 genes ranked by the coefficient of variation of their gene expression [22] derived from the Gene Expression Omnibus (http://www.ncbi.nlm.nih.gov/geo/) database. This ranking places genes along the spectrum from ubiquitously expressed to tissue-specific. The distribution of 2,003 out of 2,472 EG genes showed that essential genes fall across the continuum of gene expression (Figure S15). To assess an enrichment of essential genes for ubiquitous gene expression, we compared the overlap of essential and non-essential genes within the top 10% ubiquitously expressed genes.

### Conservation analysis

The synonymous and non-synonymous substitution rates between human and four mammalian species (rhesus, chimp, mouse and rat) were obtained from Ensembl (http://www.ensembl.org/biomart/martview/). We also obtained PhyloP [23] conservation scores for the regions (+−100 base pairs around the transcription start site) of genes from a multiple sequence alignment of 9 primate genomes from the UCSC genome browser (http://genome.ucsc.edu/).

### Statistical analysis

The statistical analyses were carried out using distribution free, non-parametric tests. All statistical tests were performed using the R language for statistical computing (http://www.r-project.org/). The significance of observed differences in characteristics of essential genes and non-essential (or all) genes was assessed using one-sided Wilcoxon Rank Sum Tests. When comparing two different properties within samples the paired test was used, otherwise tests were unpaired. 2×2 contingency tables were analyzed using the two-sided Fisher's exact test.

### Ingenuity pathway analysis

Enrichment of essential genes in functional categories was scored using the Ingenuity pathway analysis (IPA) tool (www.ingenuity.com).

### Enrichment analysis of *de novo* mutations for ASD

From the supplementary material of previously published studies [1], [30]–[32], we obtained the lists of *de novo* mutations found either in ASD probands, or family-based controls. Using the annotations also provided in these supplementary datasets, we constructed two sets of variants, in which coding mutational changes (missense, nonsense, frameshift, stoploss, del_aa, inframe) and splice sites were included, but with or without synonymous mutations. This list was then filtered against the full list of all 20,029 protein-coding genes. The most damaging type of mutation was considered for genes with multiple annotations (i.e. coding>synonymous, and was unambiguous in all cases). This resulted in a total number of *de novo* mutations in cases/controls, respectively, for each data set as: [+synonymous]: 169/- (Neale), 255/50 (O'Roak), 314/278 (Iossifov), and 171/122 (Sanders); [−synonymous]: 119/- (Neale), 188/34 (O'Roak), 234/211 (Iossifov), 141/84 (Sanders).

Because essential genes tend to have longer total exon lengths compared to genes in the genome on average, one expects more *de novo* events to occur there. Furthermore, we noted a significant correlation between %GC and number of *de novo* events (data not shown). To account for these effects, for each data set, we performed a within-study, gene-based permutation procedure that randomly exchanged for each gene in the above constructed list, another gene from the list of 20,029 with a similar exon length (within +/−100 bp) and %GC content (within +/−2.5% GC) with replacement. 2D contour distribution of total exon length with GC show there is sufficient overlap between gene sets for our exchange procedure to be appropriate (Figure S16). From this, the simulated expectation for the number of *de novo* events in essential genes was used to calculate the presented length and GC-“adjusted” odds-ratio (OR). To compute the overall effect and statistical significance, we applied inverse-variance weighted meta-analysis under a fixed effects model. No significant heterogeneity was observed in any comparison (i.e., Cochran's *Q* P-value >0.05 in all cases).

## Supporting Information

Figure S1 Results of Ingenuity Pathway Analysis of 2,472 essential genes for enrichment in a) molecular and cellular functions, b) physiological systems and development and c) diseases and disorders.
(PDF)

Figure S2 Gene expression profiles of *CSRP3* for mouse (left) and human (right) obtained from the BioGPS website. In both mouse and human there is tissue-specific expression in the heart.
(PDF)

Figure S3 Gene expression profile of *SLC12A1* for mouse (left) and human (right) obtained from the BioGPS website. In both mouse and human there is tissue-specific expression in the kidney.
(PDF)

Figure S4 Gene expression profile of *SCN3A* for mouse (left) and human (right) obtained from the BioGPS website. In both mouse and human there is tissue-specific expression in the brain.
(PDF)

Figure S5 Comparison of average sequence conservation in exonic regions in essential genes, non-essential genes and all genes. Coding regions of essential genes are significantly more conserved than in the other two groups (EG versus NLG: *P* = 1.28×10^−75^ and EG versus ALL: *P* = 1.18×10^−161^).
(PNG)

Figure S6 Comparison of average sequence conservation in promoter regions (+−100 bp around the transcription start site) in essential genes, non-essential genes and all genes. The promoter regions of essential genes are, on average, more conserved than in the other two groups (EG versus NLG: *P* = 7.93×10^−6^ and EG versus ALL: *P* = 1.71×10^−31^, Wilcoxon test).
(PNG)

Figure S7 Average, gene length corrected, numbers of exonic missense variants in EG, NLG and ALL in the 1000 Genomes dataset. The plotted Z-score is normalized relative to the Box-Cox log-transformation (i.e. pseudo-count of 1 has been added to each gene) of variant counts in all protein coding genes.
(PNG)

Figure S8 Allele frequency distributions in four continental populations (AFR, AMR, ASN, CEU) and the combined 1000 Genomes sample for the EG, NLG and ALL gene sets.
(PNG)

Figure S9 Comparison of the ratio of non-synonymous and synonymous exonic variants in EG, NLG and ALL in the 54 HapMap samples sequenced by CGI. There is a significantly lower ratio in essential genes (*P* = 8.36×10^−11^).
(PNG)

Figure S10 Comparison of the gene-length corrected average number of exonic missense variants in EG, NLG and ALL for the 54 HapMap CGI genomes. The essential genes show a significantly reduced average (*P* = 8.36×10^−11^).
(PNG)

Figure S11 Boxplot of the distribution of exonic missense variants (left), putative damaging exonic variants (center) and loss-of-function variants in the 1000 Genomes samples. The box extends from the lower to the upper quartile, the red bar indicated the median.
(PNG)

Figure S12 Protein-protein interaction network analysis generated by DAPPLE [34]. Circles denote genes and lines connecting genes indicate physical interactions. Here, only direct connections among the input genes are plotted. The colors indicate the statistical evidence for the enrichment of connectivity for an individual gene in the given network. A) Network analysis of 179 essential genes with *de novo* variants in ASD cases but not family-based controls. The significance of the overall excess of connectivity in this network is *P* = 0.0019 (114 direct connections observed, ∼84 expected). B) Network analysis of 399 essential genes with over-expression in the brain. We observe an excess of connectivity (*P* = 0.00099, 502 direct connection observed, ∼256 expected).
(EPS)

Figure S13 Comparison of the length of coding sequence (CDS) in essential, non-essential and all genes. The CDS of essential genes is on average significantly longer than for either non-essential (*P* = 2.92×10^−30^, Wilcoxon test) or all protein coding genes (*P* = 1.73×10^−92^, Wilcoxon test).
(PNG)

Figure S14 Comparison of average GC content in essential, non-essential and all protein coding genes. There is no significant difference between essential genes and the genomic average for all genes (*P* = 0.38, Wilcoxon test).
(PNG)

Figure S15 List of 13,629 genes ranked by the coefficient of variation (CV) of gene expression [9]. A small CV identifies genes with ubiquitous expression, a large CV marks tissue-specific expression. Expression signatures of essential genes (red) are spread along the entire continuum, but there is significant enrichment (Fisher's Exact *P* = 9.23×10^−21^, OR = 3.12, 95% CI 2.43–4.04) within the top 10% genes with ubiquitous expression.
(PNG)

Figure S16 2D contour distribution of total exon length and GC content for the EG, NLG and ALL gene sets. It can be seen that there is sufficient overlap between gene sets for our exchange procedure to be appropriate.
(PNG)

Table S1 Wilcoxon test P-values for comparison of non-synonymous to synonymous substitution rates in essential genes for two primate (rhesus and chimp) and two rodent (mouse and rat) species. In each case a significantly smaller ratio, i.e. an enrichment for synonymous substitution is observed for the essential genes.
(DOC)

Table S2 Contingency table for comparisons of essential with non-essential genes for four different biological properties. Differences in essential genes compared to non-essential genes are tested using Fisher's exact test for A) human disease genes (*P* = 1.73×10^−14^, OR = 1.57, 95% CI 1.39–1.77), B) haploinsufficiency (*P* = 1.75×10^−33^, OR = 4.91, 95% CI 3.69–6.62), C) ubiquitous gene expression (*P* = 9.23×10^−21^, OR = 3.12, 95% CI 2.43–4.04), D) loss-of-function variants (*P* = 0.012, OR = 0.67, 95% CI 0.48–0.92) and E) 112 known ASD candidate genes (*P* = 0.001, OR = 2.08, 95% CI 1.31–3.3).
(DOC)

Table S3 Wilcoxon test P-values for comparisons of the gene length corrected incidence of exonic missense variants in essential, non-essential and all genes. Results are given for the 14 1000 Genomes subpopulations, the four continental populations (African, American, Asian, European) and all samples. For each population a significant reduction in exonic missense variants in essential genes is observed.
(DOC)

Table S4 Wilcoxon test P-values for comparison of allele frequencies in essential, non-essential and all genes. Results are given for the 14 1000 Genomes subpopulations, the four continental populations (African, American, Asian, European) and all samples. For each population a significant shift towards rare alleles is observed in essential genes.
(DOC)

Table S5 46 lethal phenotypes identified in the MGI database.
(DOC)

Table S6 List of 20,029 protein coding genes from the CCDS database. Annotations include alternative gene names, chromosome position, essentiality status, number of exons, total length of exonic sequence and average GC content.
(XLS)

Table S7 List of 6,506 rare (<1%), putatively damaging exonic missense variants within essential genes identified in the 1000 Genomes Phase 1 data.
(XLS)

Table S8 List of 555 rare (<1%) loss-of-function variants within essential genes identified in the 1000G phase 1 data.
(XLS)

Table S9 List of 553 rare (<1%), putatively damaging exonic missense variants within essential genes identified in 54 CGI whole-genome sequences of HapMap individuals.
(XLS)

Table S10 List of 84 rare (<1%) loss-of-function variants within essential genes identified in 54 CGI whole-genome sequences of HapMap individuals.
(XLS)

Table S11 List of 179 essential genes with *de novo* variants in ASD cases and no variants in family-based controls.
(XLS)

Text S1 The supplementary methods include additional analyses of conservation of tissue-specific expression in essential genes, enrichment of alternative transcripts in essential genes and differences of essential and non-essential genes in 54 HapMap whole genome sequences.
(DOCX)
