# Supplementary figures and images for: From Mouse to Human: Evolutionary Genomics Analysis of Human Orthologs of Essential Genes
Source: PLoS Genet. 2013 May 9;9(5):e1003484. doi: 10.1371/journal.pgen.1003484 (PMC3649967; doi:10.1371/journal.pgen.1003484)

A)

■ Final 2472 EG - 2012-12-20 04:48 PM

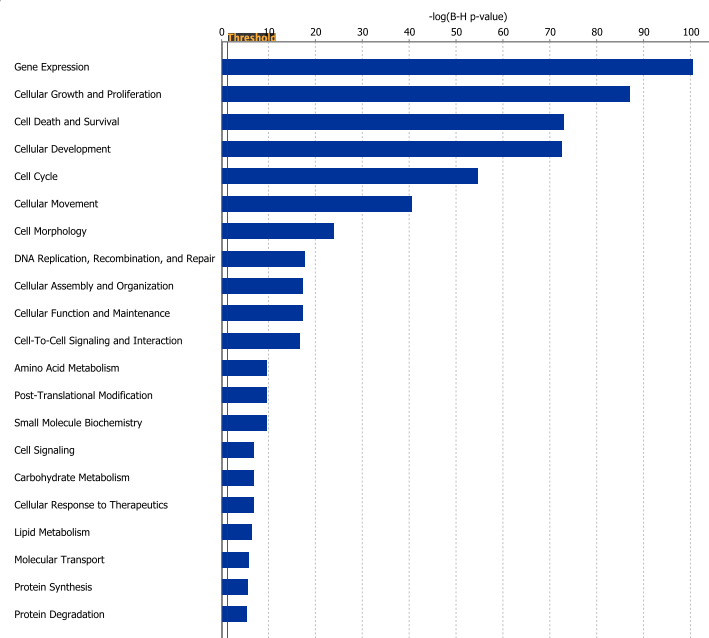

C)

■ Final 2472 EG - 2012-12-20 04:48 PM

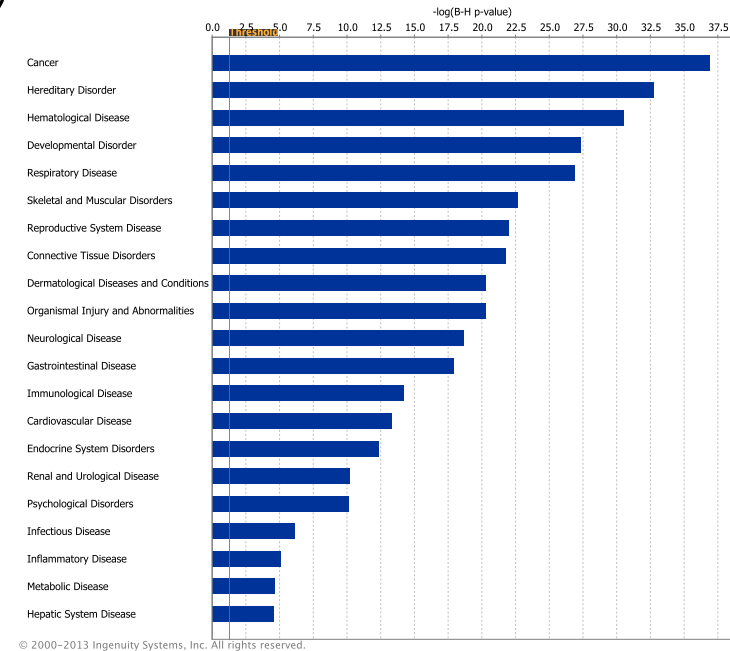

B)

■ Final 2472 EG - 2012-12-20 04:48 PM

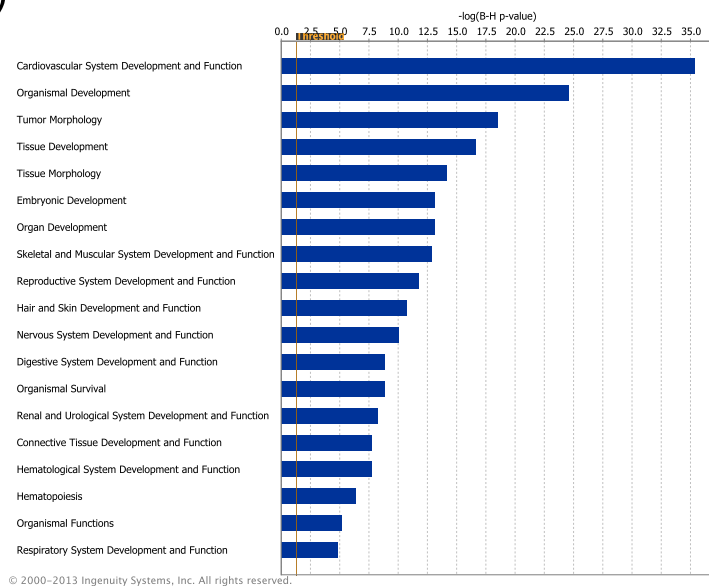

Supplement: Figure S1 — Results of Ingenuity Pathway Analysis of 2,472 essential genes for enrichment in a) molecular and cellular functions, b) physiological systems and development and c) diseases and disorders. (PDF) [file pgen.1003484.s001.pdf]

1460318\_at

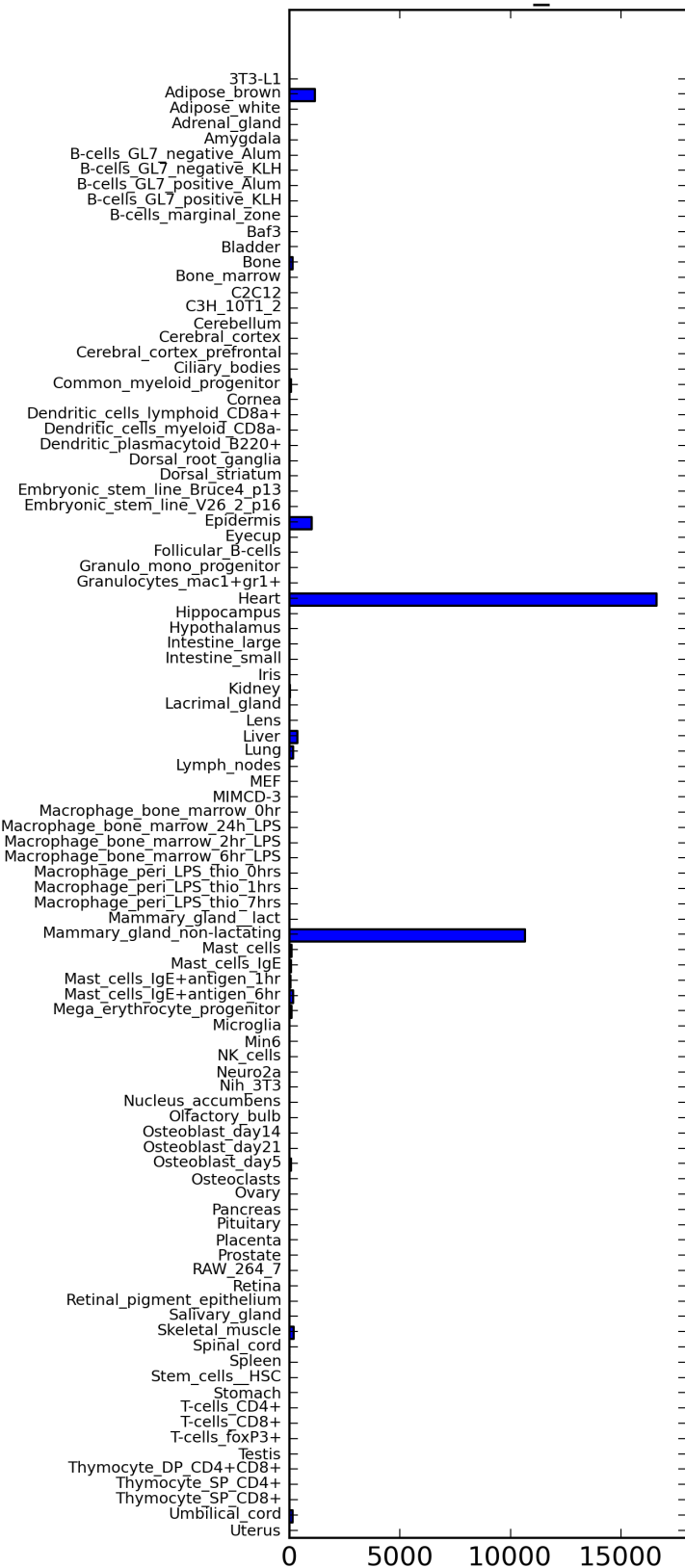

205553\_s\_at

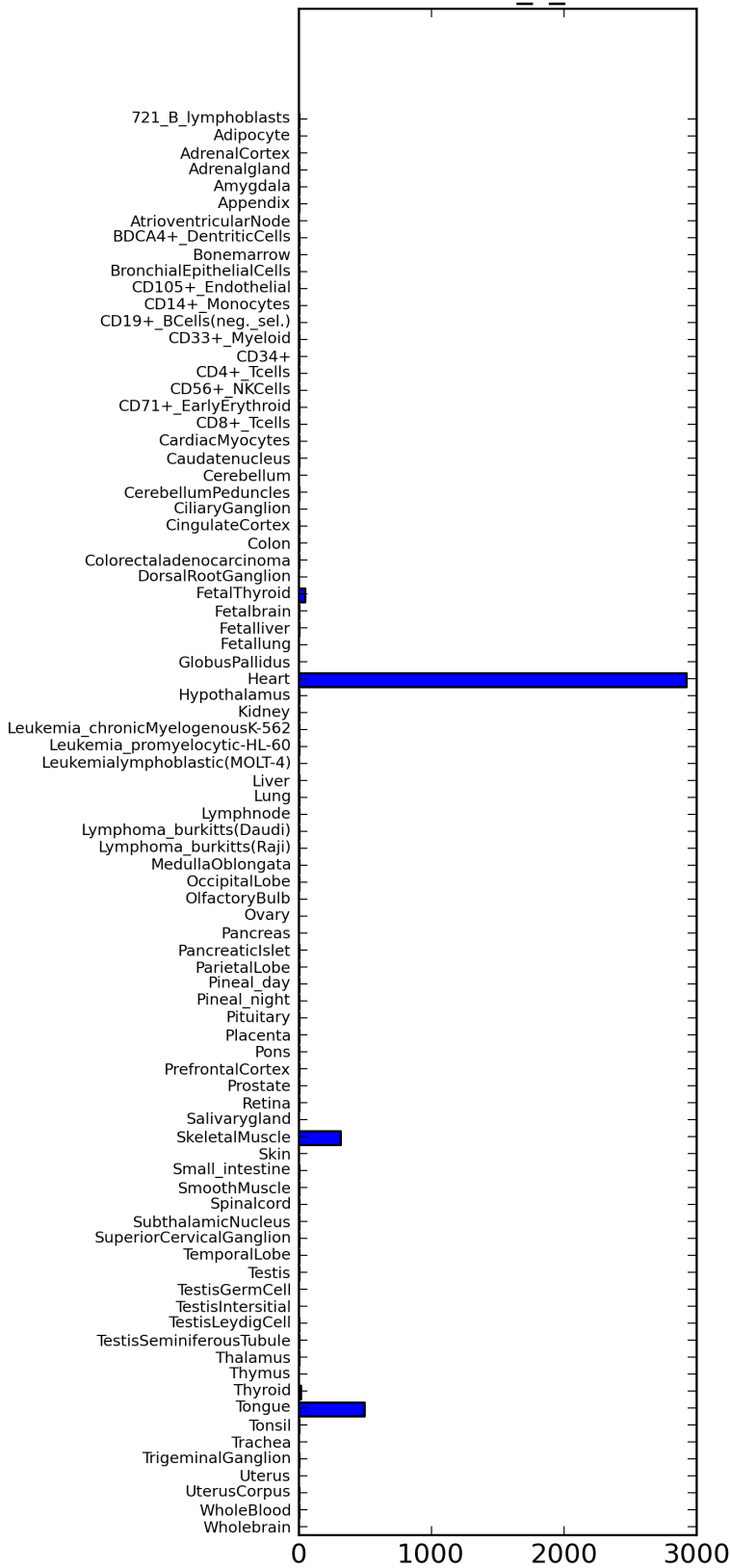

Supplement: Figure S2 — Gene expression profiles of CSRP3 for mouse (left) and human (right) obtained from the BioGPS website. In both mouse and human there is tissue-specific expression in the heart. (PDF) [file pgen.1003484.s002.pdf]

1421390\_at

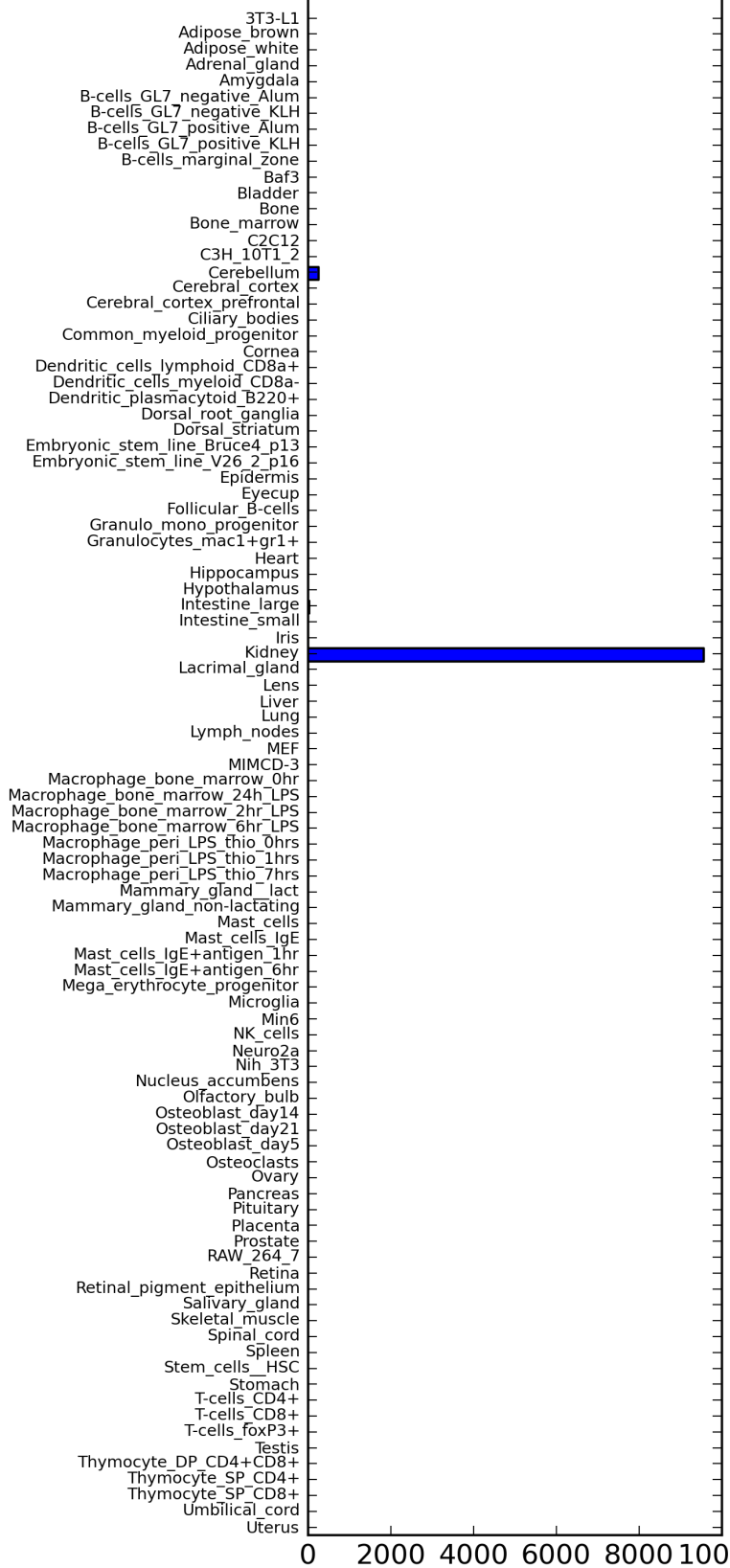

220281\_at

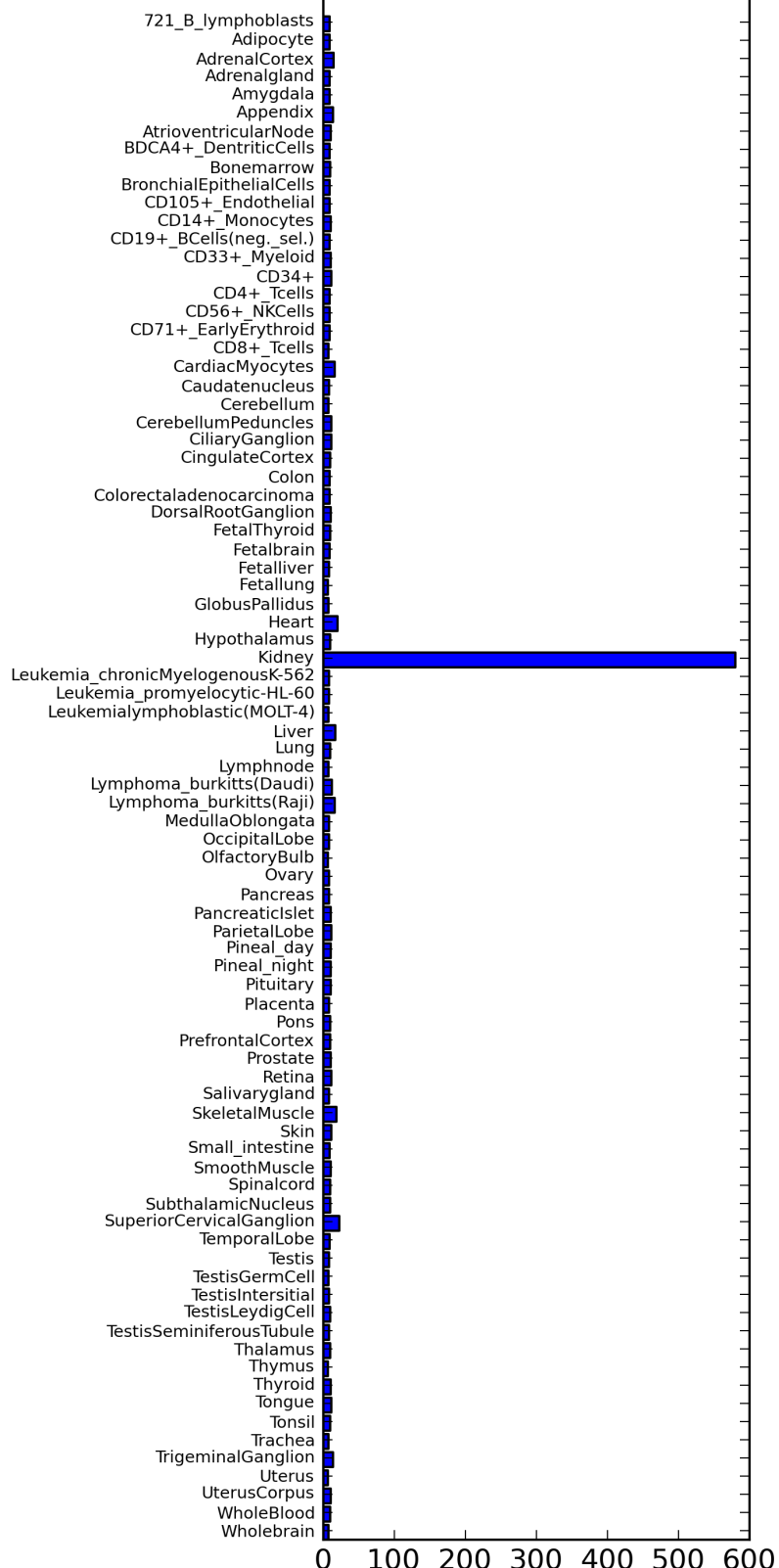

Supplement: Figure S3 — Gene expression profile of SLC12A1 for mouse (left) and human (right) obtained from the BioGPS website. In both mouse and human there is tissue-specific expression in the kidney. (PDF) [file pgen.1003484.s003.pdf]

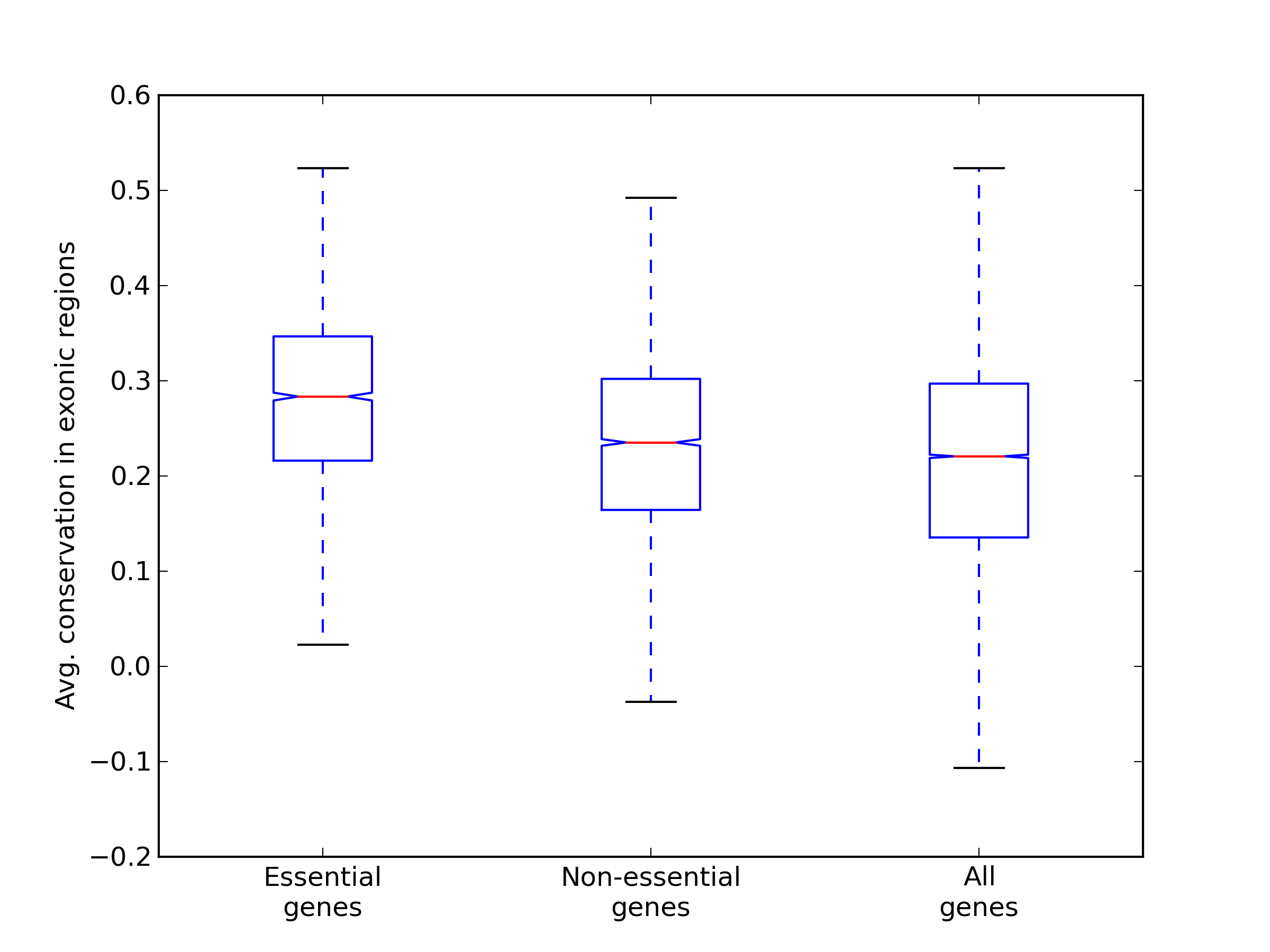

Supplement: Figure S5 — Comparison of average sequence conservation in exonic regions in essential genes, non-essential genes and all genes. Coding regions of essential genes are significantly more conserved than in the other two groups (EG versus NLG: P = 1.28×10^−75^ and EG versus ALL: P = 1.18×10−161). (PNG) [file pgen.1003484.s005.png]

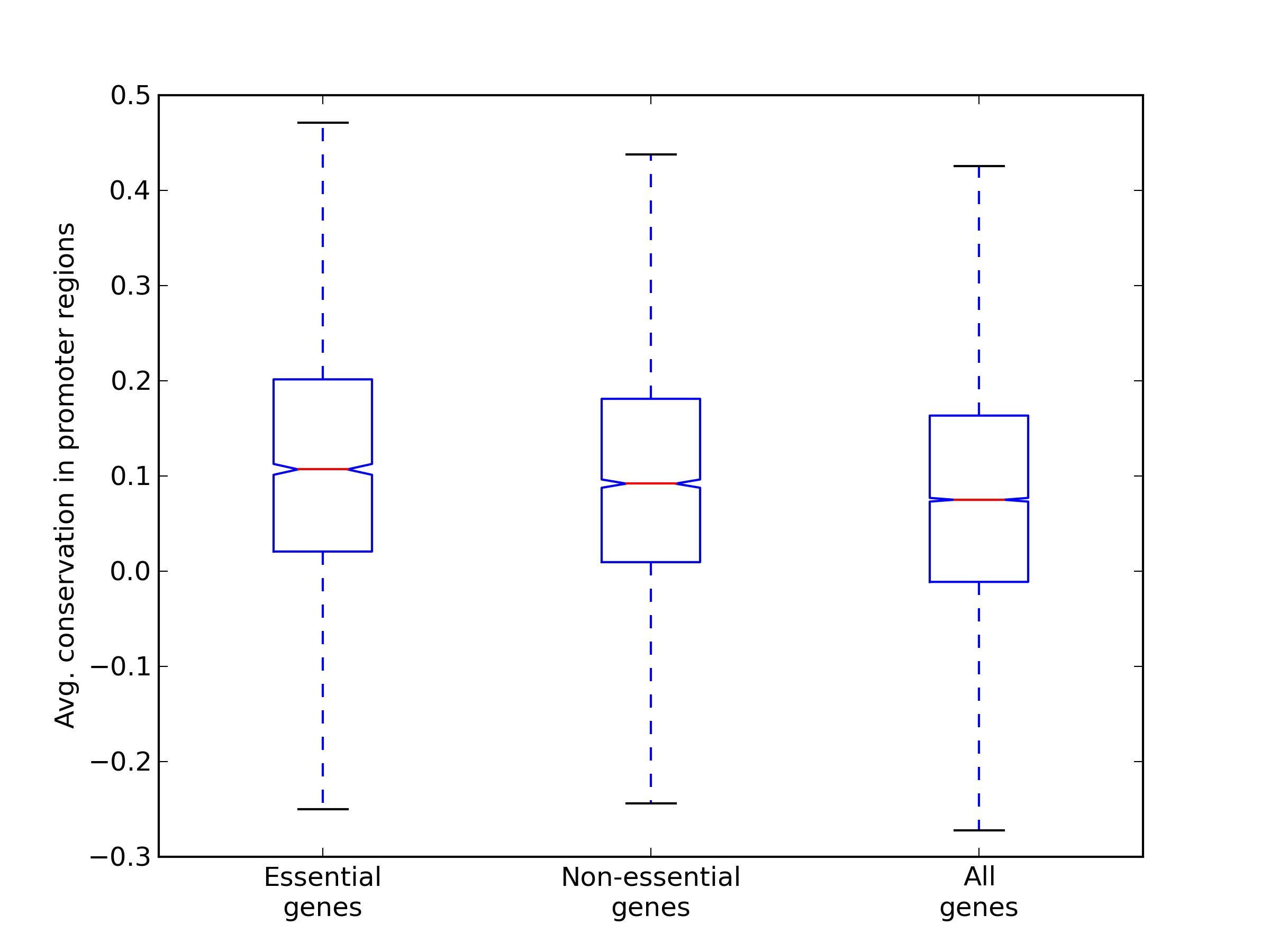

Supplement: Figure S6 — Comparison of average sequence conservation in promoter regions (+−100 bp around the transcription start site) in essential genes, non-essential genes and all genes. The promoter regions of essential genes are, on average, more conserved than in the other two groups (EG versus NLG: P = 7.93×10^−6^ and EG versus ALL: P = 1.71×10^−31^, Wilcoxon test). (PNG) [file pgen.1003484.s006.png]

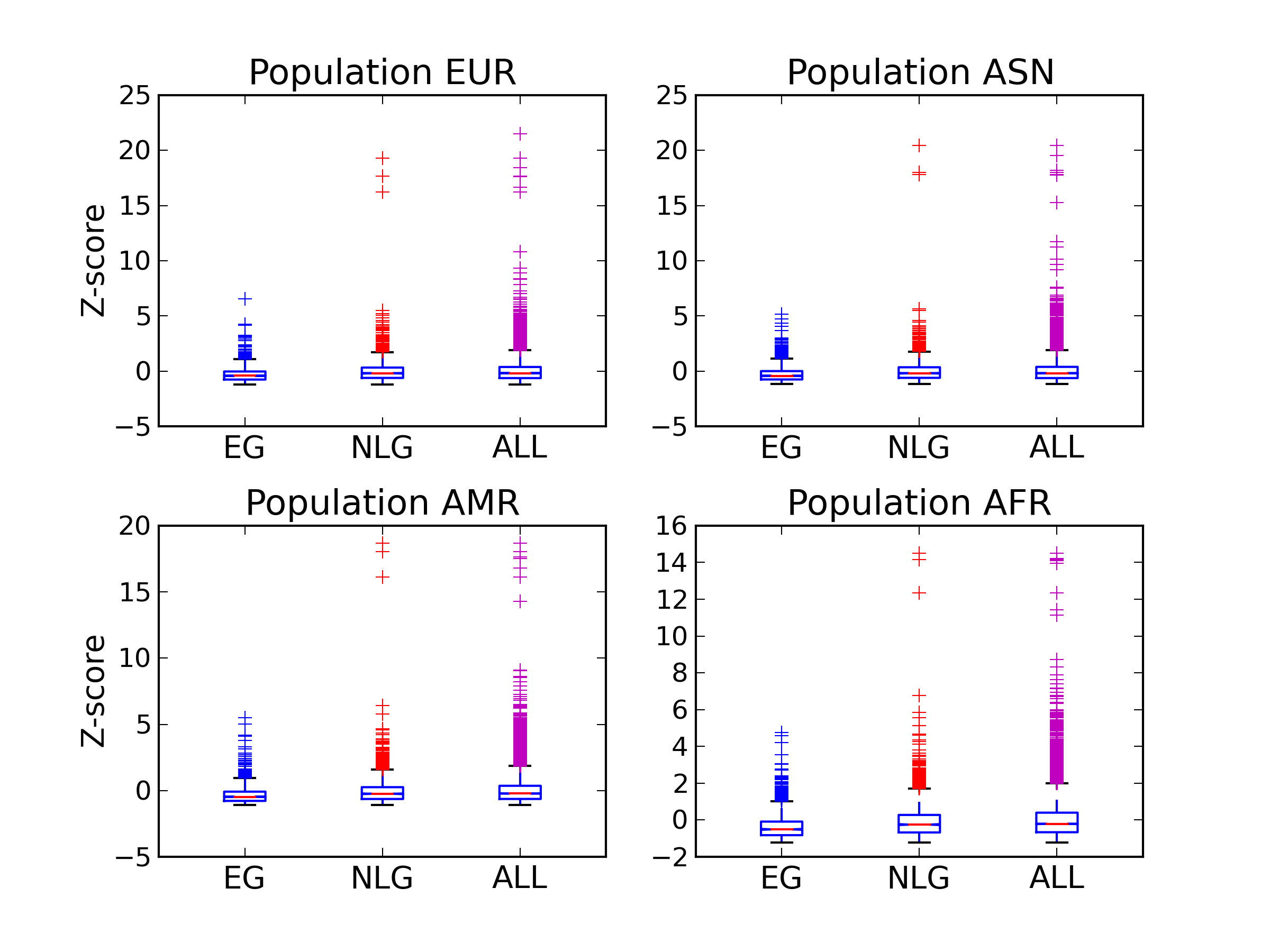

Supplement: Figure S7 — Average, gene length corrected, numbers of exonic missense variants in EG, NLG and ALL in the 1000 Genomes dataset. The plotted Z-score is normalized relative to the Box-Cox log-transformation (i.e. pseudo-count of 1 has been added to each gene) of variant counts in all protein coding genes. (PNG) [file pgen.1003484.s007.png]

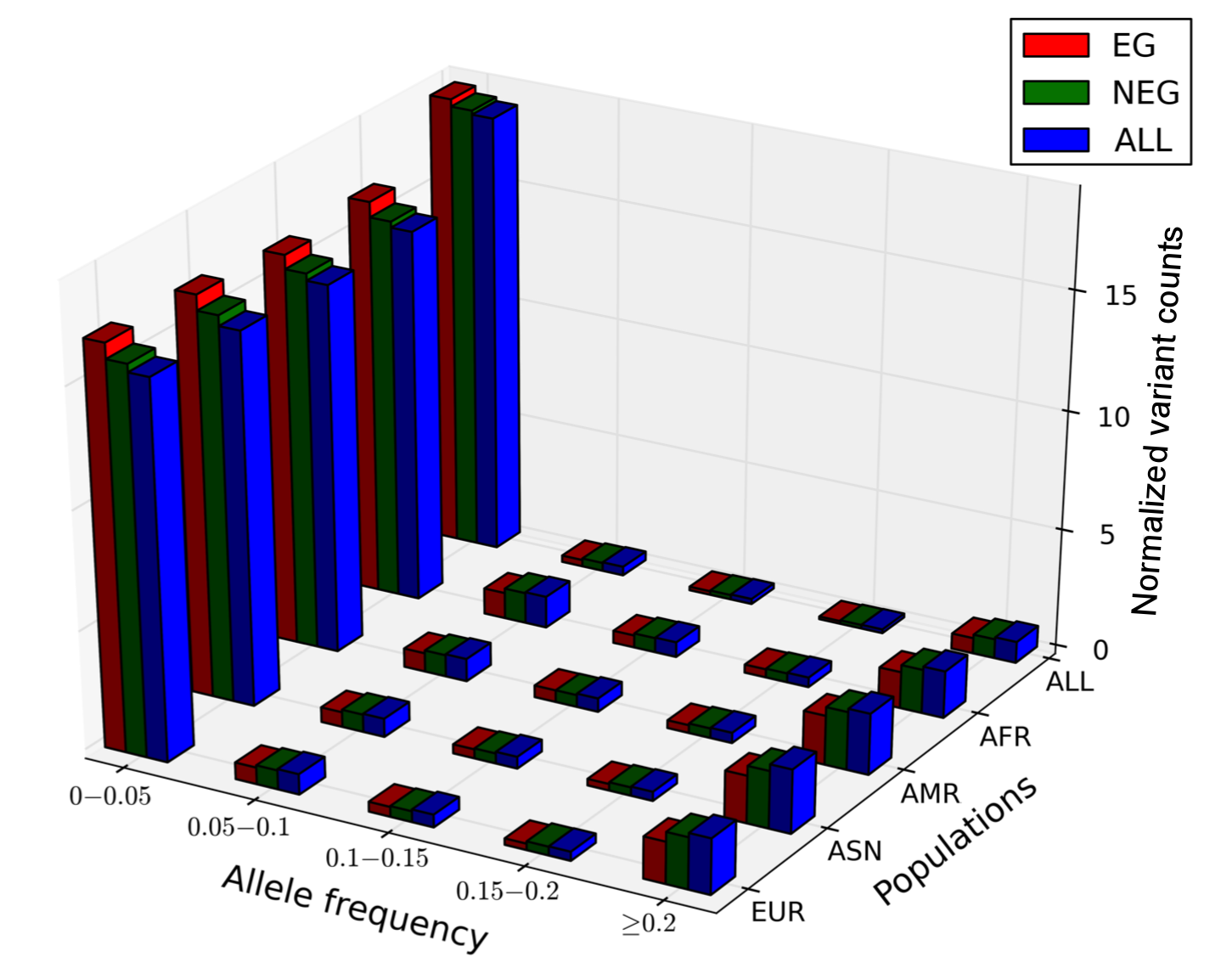

Supplement: Figure S8 — Allele frequency distributions in four continental populations (AFR, AMR, ASN, CEU) and the combined 1000 Genomes sample for the EG, NLG and ALL gene sets. (PNG) [file pgen.1003484.s008.png]

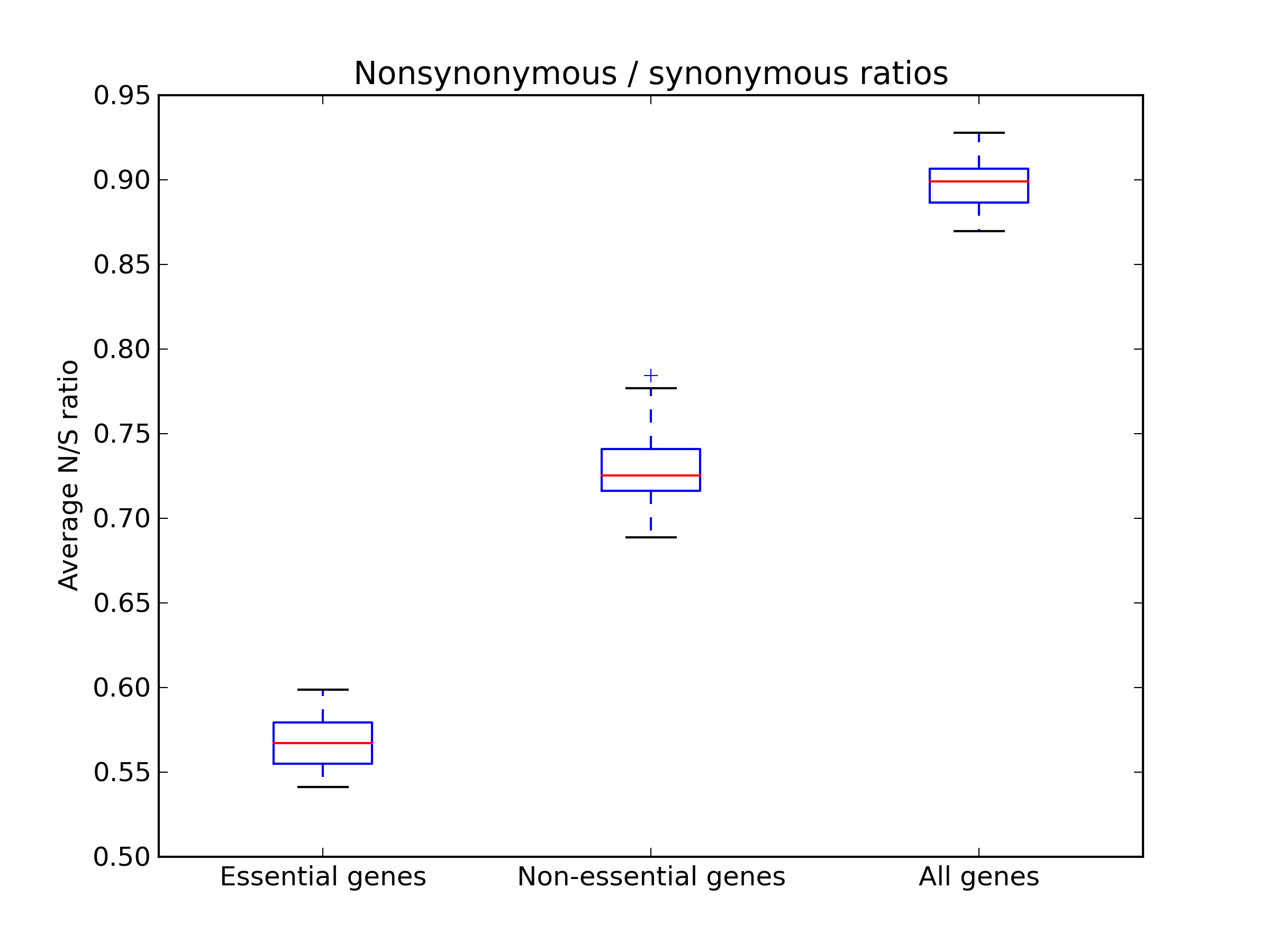

Supplement: Figure S9 — Comparison of the ratio of non-synonymous and synonymous exonic variants in EG, NLG and ALL in the 54 HapMap samples sequenced by CGI. There is a significantly lower ratio in essential genes (P = 8.36×10^−11^). (PNG) [file pgen.1003484.s009.png]

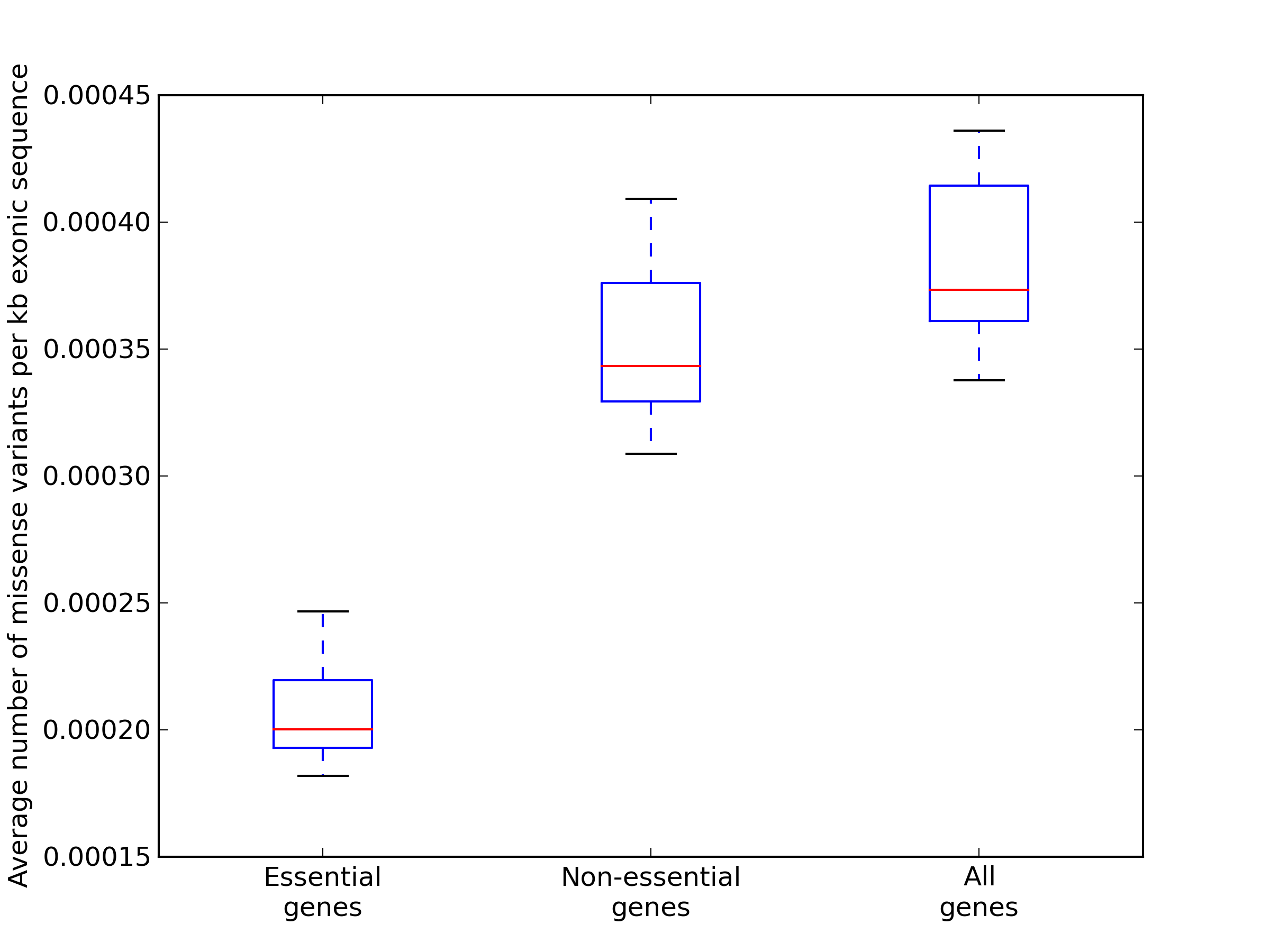

Supplement: Figure S10 — Comparison of the gene-length corrected average number of exonic missense variants in EG, NLG and ALL for the 54 HapMap CGI genomes. The essential genes show a significantly reduced average (P = 8.36×10^−11^). (PNG) [file pgen.1003484.s010.png]

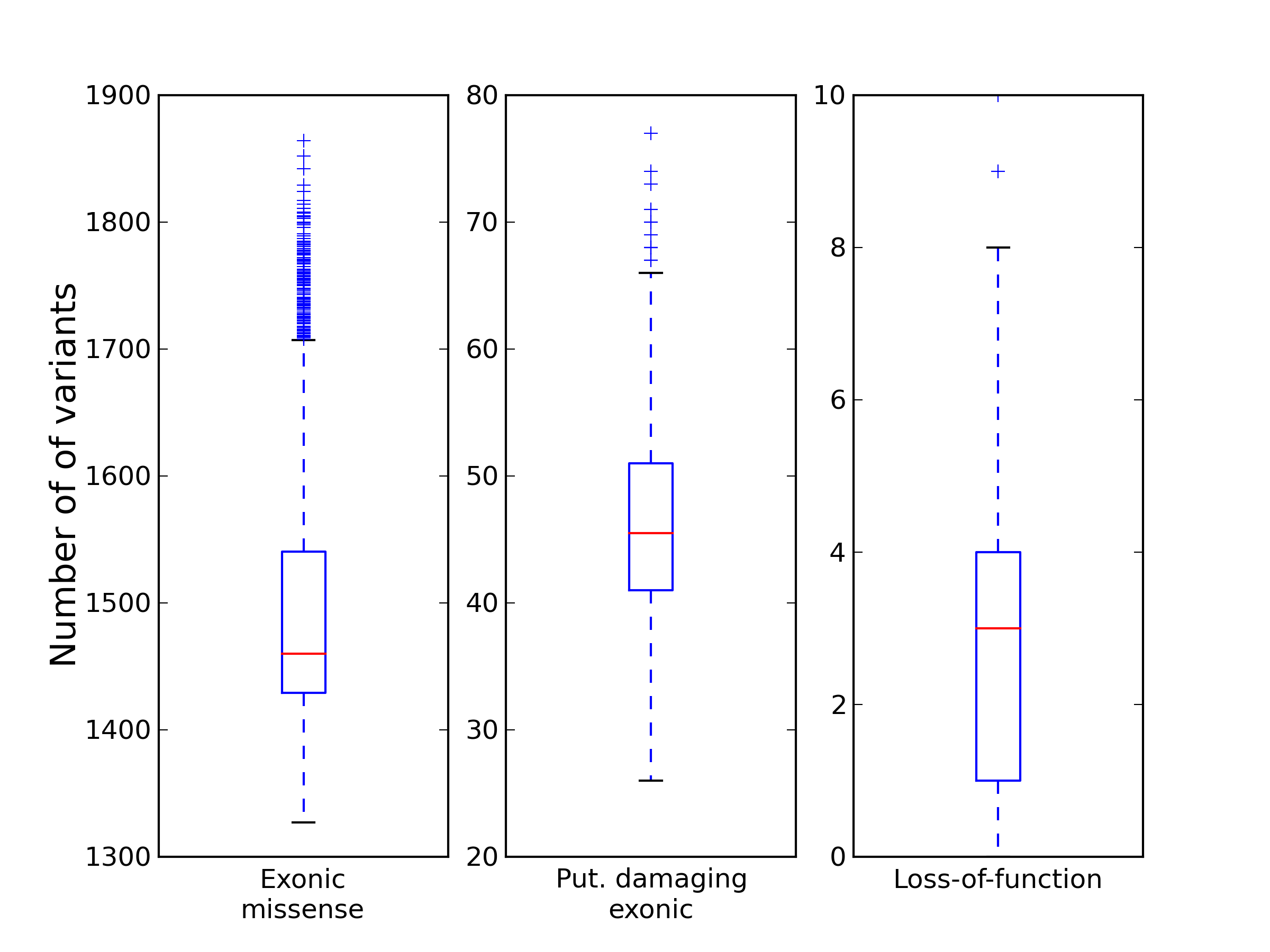

Supplement: Figure S11 — Boxplot of the distribution of exonic missense variants (left), putative damaging exonic variants (center) and loss-of-function variants in the 1000 Genomes samples. The box extends from the lower to the upper quartile, the red bar indicated the median. (PNG) [file pgen.1003484.s011.png]

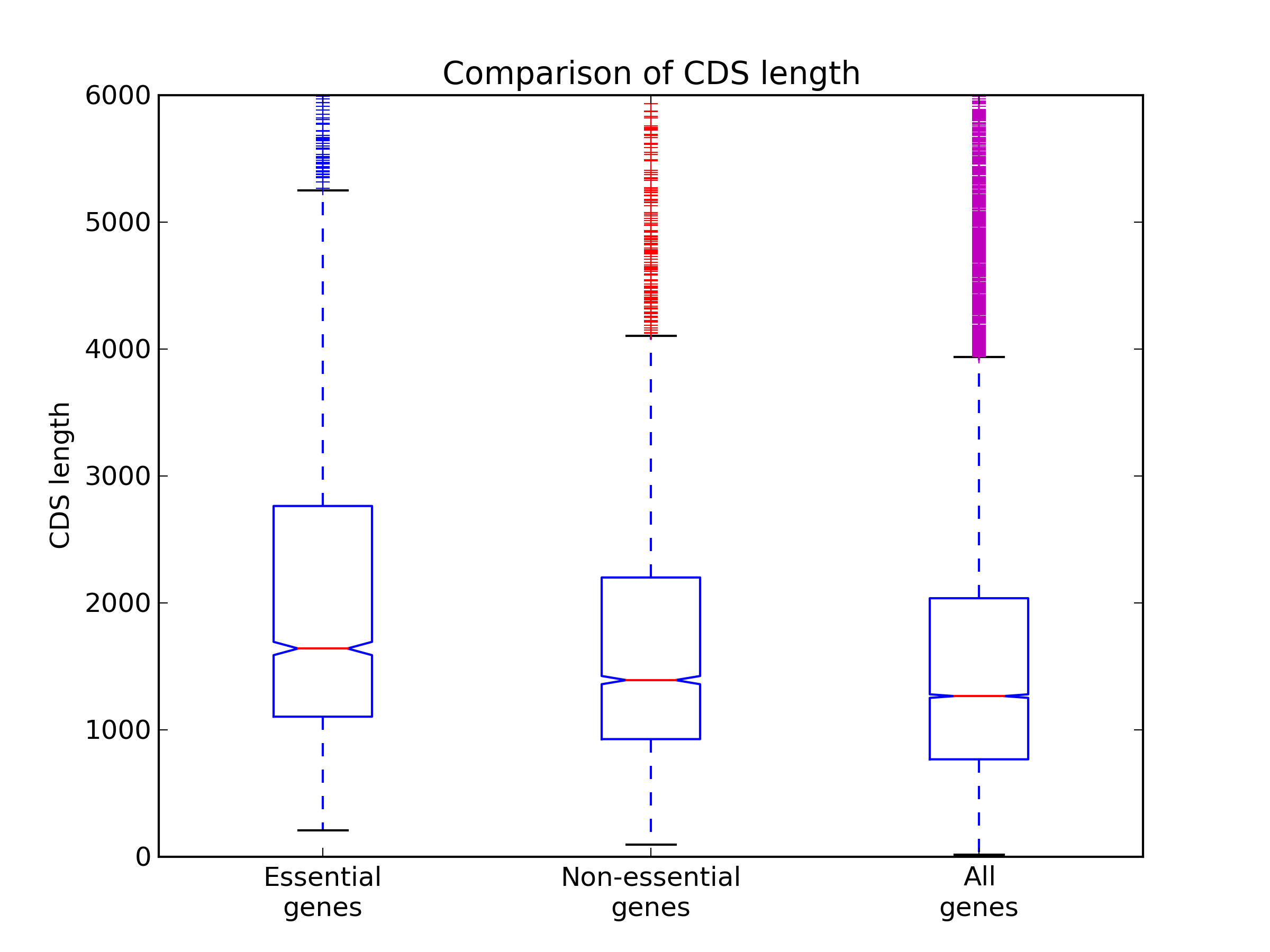

Supplement: Figure S13 — Comparison of the length of coding sequence (CDS) in essential, non-essential and all genes. The CDS of essential genes is on average significantly longer than for either non-essential (P = 2.92×10^−30^, Wilcoxon test) or all protein coding genes (P = 1.73×10^−92^, Wilcoxon test). (PNG) [file pgen.1003484.s013.png]

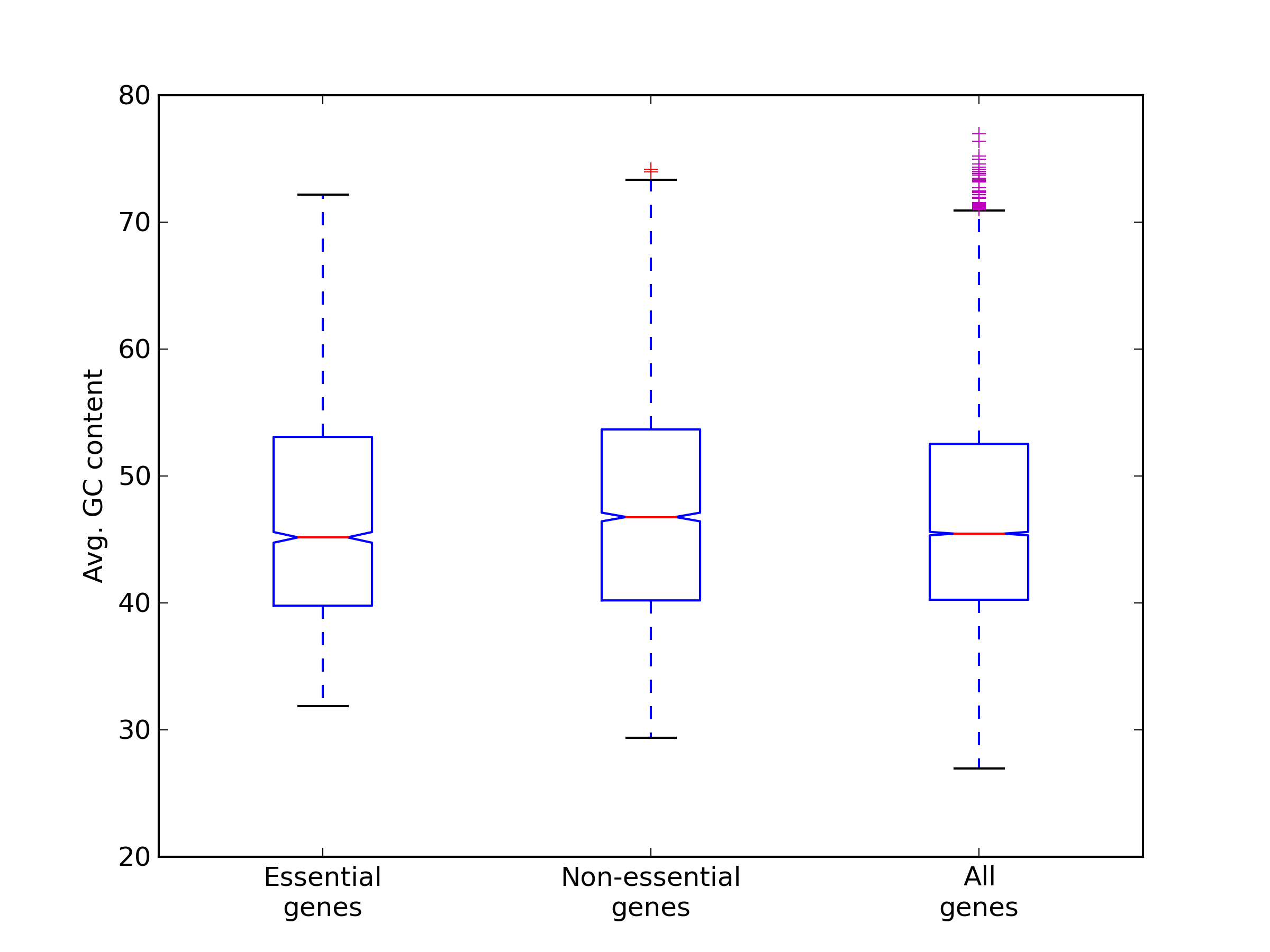

Supplement: Figure S14 — Comparison of average GC content in essential, non-essential and all protein coding genes. There is no significant difference between essential genes and the genomic average for all genes (P = 0.38, Wilcoxon test). (PNG) [file pgen.1003484.s014.png]

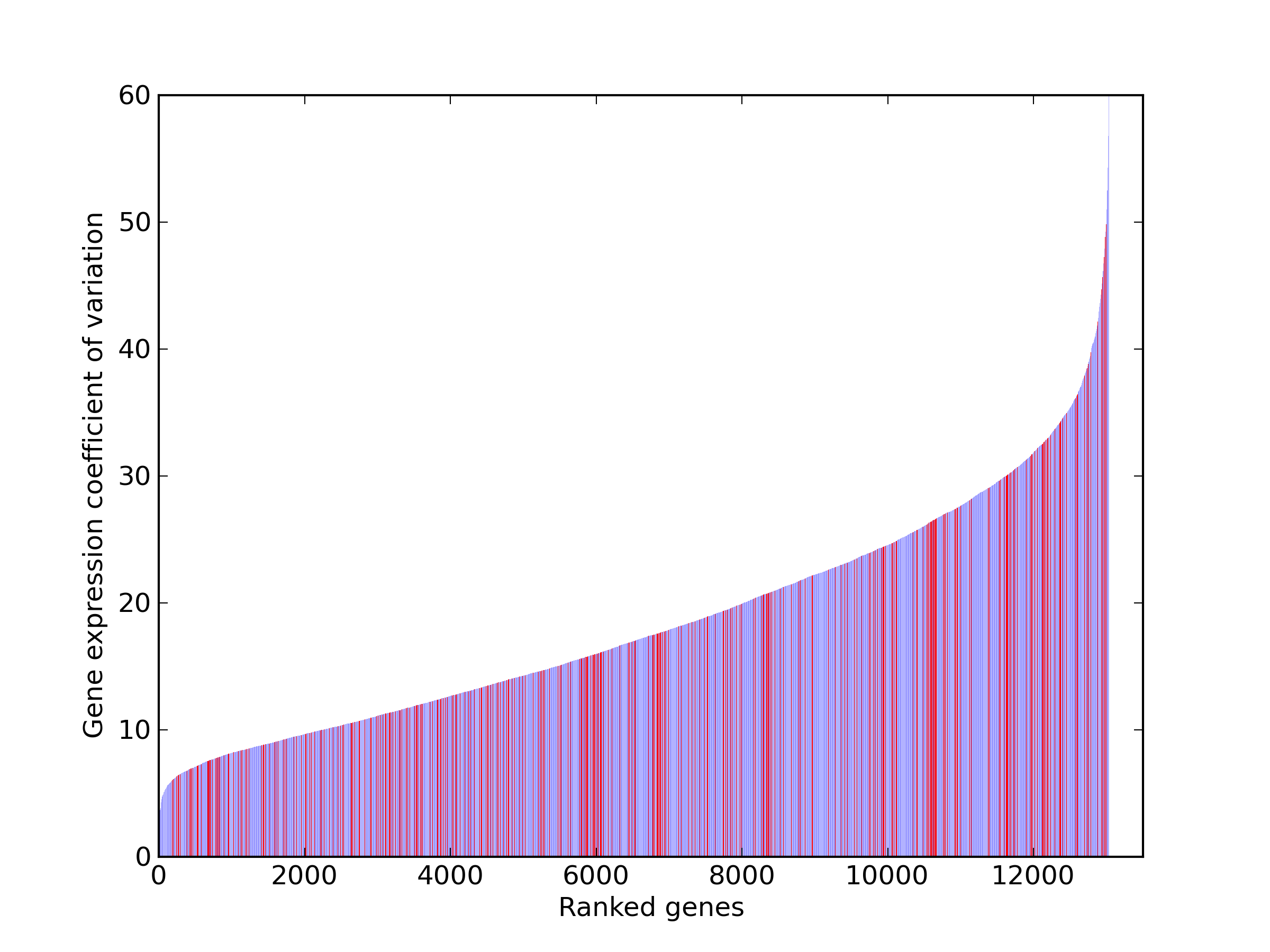

Supplement: Figure S15 — List of 13,629 genes ranked by the coefficient of variation (CV) of gene expression [9]. A small CV identifies genes with ubiquitous expression, a large CV marks tissue-specific expression. Expression signatures of essential genes (red) are spread along the entire continuum, but there is significant enrichment (Fisher's Exact P = 9.23×10^−21^, OR = 3.12, 95% CI 2.43–4.04) within the top 10% genes with ubiquitous expression. (PNG) [file pgen.1003484.s015.png]

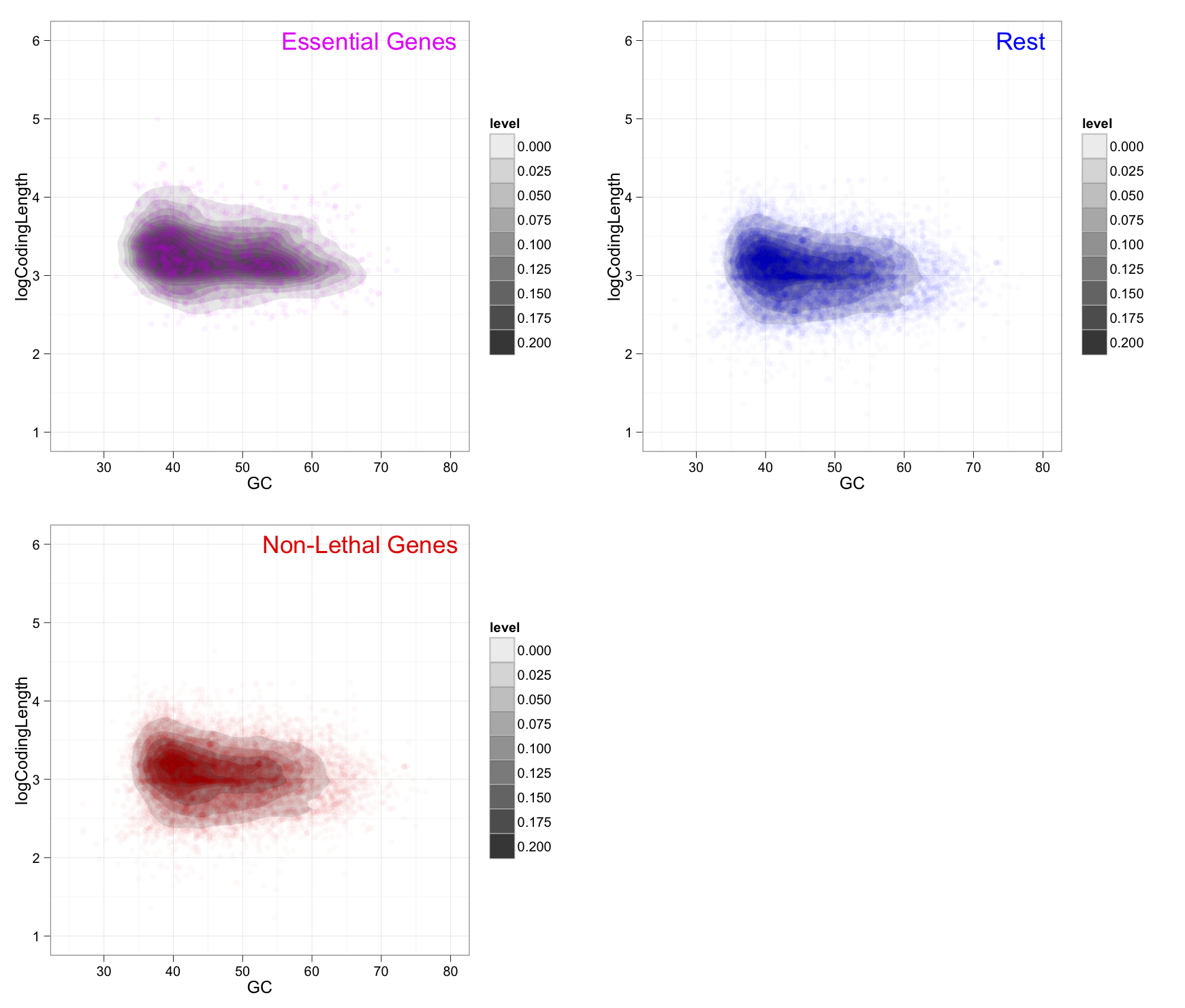

Supplement: Figure S16 — 2D contour distribution of total exon length and GC content for the EG, NLG and ALL gene sets. It can be seen that there is sufficient overlap between gene sets for our exchange procedure to be appropriate. (PNG) [file pgen.1003484.s016.png]
