# Supplementary material for: From Mouse to Human: Evolutionary Genomics Analysis of Human Orthologs of Essential Genes
Source: PLoS Genet. 2013 May 9;9(5):e1003484. doi: 10.1371/journal.pgen.1003484 (PMC3649967; doi:10.1371/journal.pgen.1003484)
Supplement: Table S1 — Wilcoxon test P-values for comparison of non-synonymous to synonymous substitution rates in essential genes for two primate (rhesus and chimp) and two rodent (mouse and rat) species. In each case a significantly smaller ratio, i.e. an enrichment for synonymous substitution is observed for the essential genes. (DOC) [file pgen.1003484.s017.doc]

| Species | dN/dS EG vs NLG | dN/dS EG vs ALL |
| --- | --- | --- |
| Rhesus | 5.15 x 10^-34^ | 8.96 x 10^-63^ |
| Chimp | 2.08 x 10^-29^ | 7.02 x 10^-67^ |
| Mouse | 5.39 x 10^-72^ | 1.72 x 10^-98^ |
| Rat | 1.38 x 10^-66^ | 8.26 x 10^-93^ |
