# Supplementary material for: From Mouse to Human: Evolutionary Genomics Analysis of Human Orthologs of Essential Genes
Source: PLoS Genet. 2013 May 9;9(5):e1003484. doi: 10.1371/journal.pgen.1003484 (PMC3649967; doi:10.1371/journal.pgen.1003484)
Supplement: Table S2 — Contingency table for comparisons of essential with non-essential genes for four different biological properties. Differences in essential genes compared to non-essential genes are tested using Fisher's exact test for A) human disease genes (P = 1.73×10^−14^, OR = 1.57, 95% CI 1.39–1.77), B) haploinsufficiency (P = 1.75×10^−33^, OR = 4.91, 95% CI 3.69–6.62), C) ubiquitous gene expression (P = 9.23×10^−21^, OR = 3.12, 95% CI 2.43–4.04), D) loss-of-function variants (P = 0.012, OR = 0.67, 95% CI 0.48–0.92) and E) 112 known ASD candidate genes (P = 0.001, OR = 2.08, 95% CI 1.31–3.3). (DOC) [file pgen.1003484.s018.doc]

| A) |  | HGMD | not HGMD |
| --- | --- | --- | --- |
|  | Essential | 758 | 1714 |
|  | Non-essential | 837 | 2974 |
| B) |  | Haploinsufficient | Not haploinsufficient |
|  | Essential | 200 | 2272 |
|  | Non-essential | 67 | 3744 |
| C) |  | Top 10% ubiquitous exp. | Other genes |
|  | Essential | 192 | 2280 |
|  | Non-essential | 100 | 3711 |
| D) |  | LoF variants | No LoF variants |
|  | Essential | 58 | 2414 |
|  | Non-essential | 132 | 3679 |
| E) |  | ASD candidate | Not ASD candidate |
|  | Essential | 48 | 2424 |
|  | Non-essential | 36 | 3775 |
