# Supplementary material for: From Mouse to Human: Evolutionary Genomics Analysis of Human Orthologs of Essential Genes
Source: PLoS Genet. 2013 May 9;9(5):e1003484. doi: 10.1371/journal.pgen.1003484 (PMC3649967; doi:10.1371/journal.pgen.1003484)
Supplement: Table S3 — Wilcoxon test P-values for comparisons of the gene length corrected incidence of exonic missense variants in essential, non-essential and all genes. Results are given for the 14 1000 Genomes subpopulations, the four continental populations (African, American, Asian, European) and all samples. For each population a significant reduction in exonic missense variants in essential genes is observed. (DOC) [file pgen.1003484.s019.doc]

| 1000G Population | Missense variants  (EG vs NLG) | Missense variants  (EG vs ALL) | Missense variants  (NLG vs ALL) |
| --- | --- | --- | --- |
| Colombians (CLM) | 3.63 x 10^-33^ | 2.08 x 10^-63^ | 0.0001269 |
| Han Chinese (CHB) | 2.73 x 10^-29^ | 3.04 x 10^-48^ | 0.023 |
| Americans of African Ancestry (ASW) | 2.26 x 10^-41^ | 6.45 x 10^-79^ | 9.61 x 10^-6^ |
| Yoruba, Nigera (YRI) | 2.54 x 10^-45^ | 4.58 x 10^-83^ | 2.55 x 10^-5^ |
| Toscani, Italy (TSI) | 2.09 x 10^-36^ | 3.37 x 10^-57^ | 0.0382 |
| Mexican Ancestry (MXL) | 7.09 x 10^-38^ | 1.89 x 10^-64^ | 0.00269 |
| Luhya, Kenia (LWK) | 6.43 x 10^-44^ | 9.07 x 10^-83^ | 1.05 x 10^-5^ |
| Utah Residents, European ancestry (CEU) | 2.36 x 10^-29^ | 5.84 x 10^-53^ | 0.001649 |
| Southern Han Chinese (CHS) | 6.98 x 10^-29^ | 9.09 x 10^-46^ | 0.0536 |
| British in England and Scotland (GBR) | 6.55 x 10^-35^ | 9.29 x 10^-55^ | 0.0418 |
| Iberian population, Spain (IBS) | 1.93 x 10^-20^ | 6.37 x 10^-37^ | 0.00365 |
| Finnish (FIN) | 1.31 x 10^-28^ | 2.33 x 10^-49^ | 0.00415 |
| Japanese (JPT) | 2.34 x 10^-30^ | 2.4 x 10^-48^ | 0.0228 |
| Puerto Ricans (PUR) | 3.08 x 10^-35^ | 3.31 x 10^-62^ | 0.000854 |
| African (AFR) | 1.92 x 10^-50^ | 5.32 x 10^-90^ | 0.00018 |
| American (AMR) | 1.94 x 10^-46^ | 2.4 x 10^-78^ | 0.0019 |
| Asian (ASN) | 5.53 x 10^-38^ | 5.11 x 10e-52 | 0.423 |
| European (EUR) | 2.21 x 10^-44^ | 2.49 x 10^-64^ | 0.198 |
| All | 1.08 x 10^-59^ | 1.56 x 10e-84 | 0.309 |
