# Supplementary material for: From Mouse to Human: Evolutionary Genomics Analysis of Human Orthologs of Essential Genes
Source: PLoS Genet. 2013 May 9;9(5):e1003484. doi: 10.1371/journal.pgen.1003484 (PMC3649967; doi:10.1371/journal.pgen.1003484)
Supplement: Table S4 — Wilcoxon test P-values for comparison of allele frequencies in essential, non-essential and all genes. Results are given for the 14 1000 Genomes subpopulations, the four continental populations (African, American, Asian, European) and all samples. For each population a significant shift towards rare alleles is observed in essential genes. (DOC) [file pgen.1003484.s020.doc]

| 1000G Population | Allele frequencies  (EG vs NLG) | Allele frequencies (EG vs ALL) | Allele frequencies (NLG vs ALL) |
| --- | --- | --- | --- |
| Colombians (CLM) | 4.78 x 10^-10^ | 1.38 x 10^-23^ | 0.000567 |
| Han Chinese (CHB) | 2.26 x 10^-13^ | 2.52 x 10^-43^ | 3.04 x 10^-10^ |
| Americans of African Ancestry (ASW) | 8.88 x 10^-18^ | 9.67 x 10^-38^ | 0.00082 |
| Yoruba, Nigera (YRI) | 1.6 x 10^-16^ | 1.34 x 10^-39^ | 2.47 x 10^-5^ |
| Toscani, Italy (TSI) | 5.91 x 10^-12^ | 1.53 x 10^-38^ | 3.89 x 10^-9^ |
| Mexican Ancestry (MXL) | 4.17 x 10^-10^ | 2.68 x 10^-24^ | 0.00031 |
| Luhya, Kenia (LWK) | 1.42 x 10^-13^ | 6.35 x 10^-37^ | 1.95 x 10^-6^ |
| Utah Residents, European ancestry (CEU) | 5.78 x 10^-15^ | 9.79 x 10^-37^ | 2.77 x 10^-5^ |
| Southern Han Chinese (CHS) | 6.39 x 10^-14^ | 5.03 x 10^-41^ | 3.04 x 10^-8^ |
| British in England and Scotland (GBR) | 5.76 x 10^-10^ | 4.66 x 10^-32^ | 1.99 x 10^-8^ |
| Iberian population, Spain (IBS) | 0.00566 | 9.75 x 10^-6^ | 0.0574 |
| Finnish (FIN) | 1.68 x 10^-12^ | 2.89 x 10^-31^ | 5.51 x 10^-5^ |
| Japanese (JPT) | 3.54 x 10^-10^ | 7.97 x 10^-36^ | 7.04 x 10^-10^ |
| Puerto Ricans (PUR) | 4.25 x 10^-10^ | 1.67 x 10^-23^ | 0.000695 |
| African (AFR) | 1.63 x 10^-21^ | 3.7 x 10^-67^ | 1.18 x 10^-12^ |
| American (AMR) | 7.85 x 10^-18^ | 2.71 x 10^-44^ | 3.38 x 10^-6^ |
| Asian (ASN) | 2.37 x 10^-17^ | 4.29 x 10^-63^ | 1.23 x 10^-15^ |
| European (EUR) | 1.46 x 10^-19^ | 3.63 x 10^-62^ | 2.37 x 10^-12^ |
| All | 3.12 x 10^-35^ | 7.29 x 10^-121^ | 7.66 x 10^-24^ |
