# Supplementary material for: From Mouse to Human: Evolutionary Genomics Analysis of Human Orthologs of Essential Genes
Source: PLoS Genet. 2013 May 9;9(5):e1003484. doi: 10.1371/journal.pgen.1003484 (PMC3649967; doi:10.1371/journal.pgen.1003484)
Supplement: Table S5 — 46 lethal phenotypes identified in the MGI database. (DOC) [file pgen.1003484.s021.doc]

| MGI Phenotype | Short description |
| --- | --- |
| MP:0002058 | neonatal lethality |
| MP:0002080 | prenatal lethality |
| MP:0002081 | perinatal lethality |
| MP:0002082 | postnatal lethality |
| MP:0006204 | embryonic lethality before implantation |
| MP:0006205 | embryonic lethality before somite formation |
| MP:0006206 | embryonic lethality before turning of embryo |
| MP:0006207 | embryonic lethality during organogenesis |
| MP:0006208 | lethality throughout fetal growth and development |
| MP:0008527 | embryonic lethality at implantation |
| MP:0008569 | lethality at weaning |
| MP:0008762 | embryonic lethality |
| MP:0009850 | embryonic lethality between implantation and placentation |
| MP:0010770 | preweaning lethality |
| MP:0010832 | lethality during fetal growth through weaning |
| MP:0011083 | complete lethality at weaning |
| MP:0011084 | partial lethality at weaning |
| MP:0011085 | complete postnatal lethality |
| MP:0011086 | partial postnatal lethality |
| MP:0011087 | complete neonatal lethality |
| MP:0011088 | partial neonatal lethality |
| MP:0011089 | complete perinatal lethality |
| MP:0011090 | partial perinatal lethality |
| MP:0011091 | complete prenatal lethality |
| MP:0011092 | complete embryonic lethality |
| MP:0011093 | complete embryonic lethality at implantation |
| MP:0011094 | complete embryonic lethality before implantation |
| MP:0011095 | complete embryonic lethality between implantation and placentation |
| MP:0011096 | complete embryonic lethality before somite formation |
| MP:0011097 | complete embryonic lethality before turning of embryo |
| MP:0011098 | complete embryonic lethality during organogenesis |
| MP:0011099 | complete lethality throughout fetal growth and development |
| MP:0011100 | complete preweaning lethality |
| MP:0011101 | partial prenatal lethality |
| MP:0011102 | partial embryonic lethality |
| MP:0011103 | partial embryonic lethality at implantation |
| MP:0011104 | partial embryonic lethality before implantation |
| MP:0011105 | partial embryonic lethality between implantation and placentation |
| MP:0011106 | partial embryonic lethality before somite formation |
| MP:0011107 | partial embryonic lethality before turning of embryo |
| MP:0011108 | partial embryonic lethality during organogenesis |
| MP:0011109 | partial lethality throughout fetal growth and development |
| MP:0011110 | partial preweaning lethality |
| MP:0011111 | complete lethality during fetal growth through weaning |
| MP:0011112 | partial lethality during fetal growth through weaning |
| MP:0011400 | complete lethality |
