# Supplementary material for: From Mouse to Human: Evolutionary Genomics Analysis of Human Orthologs of Essential Genes
Source: PLoS Genet. 2013 May 9;9(5):e1003484. doi: 10.1371/journal.pgen.1003484 (PMC3649967; doi:10.1371/journal.pgen.1003484)
Supplement: Text S1 — The supplementary methods include additional analyses of conservation of tissue-specific expression in essential genes, enrichment of alternative transcripts in essential genes and differences of essential and non-essential genes in 54 HapMap whole genome sequences. (DOCX) [file pgen.1003484.s028.docx]

**From mouse to human: evolutionary genomics analysis of human orthologs of essential genes**

**Authors:** Benjamin Georgi, Benjamin F. Voight & Maja Bucan

**Text S1**

## Table of contents

Table of contents 1

Alternative transcripts in essential genes 1

Whole-genome sequence data for 54 HapMap individuals 2

Conservation of differential gene expression in essential genes between human and mouse 4

Additional references: 4

##

## Alternative transcripts in essential genes

When comparing the number of annotated transcripts for essential and non-essential genes, we find that essential genes have significantly more transcripts (Wilcoxon *P*= 2.19 x 10^-23^). To address the question whether this might introduce a bias into our analyses, we constructed equally sized subsets (N=2399) of the essential and non-essential gene sets such that the distribution of the number of transcripts was exactly matched using the following simple algorithm:

**Given**: gene set *EG* and *NLG*

**Target**: *EG_M_*, *NLG_M_*, subsets of *EG* and *NLG* with matching distributions of the number of transcipts

- For each number of transcripts *N:*
  - EG_N_ = subset of EG with *N* transcripts
  - NLG_N_ = subset of NLG with *N* transcripts
  - if | EG_N_ | >= | NLG_N_ |
    - randomly select | NLG_N_ | genes from EG_N_ and add to *EG_M_*
    - add NLG_N_ to *NLG_M_*
  - elif | EG_N_ | < | NLG_N_ |
    - add EG_N_ to *EG_M_*
    - randomly select | EG_N_ | genes from NLG_N_ and add to *NLG_M_*

We then analyzed conservation in exons and dN/dS ratios in these new, distribution matched sets and observed increased conservation in exons (Wilcoxon P= 2.62e-50) and a reduction in dN/dS ratios (Wilcoxon p =1.68e-55), confirming the results obtained with the original sets. This shows that the higher average number of transcripts in essential genes does not explain the observed increase in purifying selection.

## Whole-genome sequence data for 54 HapMap individuals

Complete Genomics (CGI) performed whole genome sequencing of 69 HapMap individuals using a sequencing-by-ligation method [29] with an average coverage of 80x and the data was released into the public domain ([ftp.completegenomics.com](ftp://ftp.completegenomics.com)). In addition to 46 unrelated subjects the data is comprised of two trios (PUR and YRI) and a three-generation, 17-member pedigree (CEPH pedigree 1463). Out of these 69 samples, we retained 54 unrelated samples from 12 populations for the analysis. Variant calls and annotations were generated and mapped to NCBI genome reference build 37 by CGI using version 2.0.0.26 of their annotation pipeline. We focused our analyses on coding variants. Functional impact was predicted using two widely-used algorithms: Polyphen2 [12] and SIFT [11]. For Polyphen2, amino acid substitutions and protein IDs for exonic missense variants were extracted from the variants files. Protein sequences were retrieved from the NCBI sequence server (<http://www.ncbi.nlm.nih.gov/sviewer/>). Substitution and protein sequences were then submitted to the Polyphen2 batch query system (<http://genetics.bwh.harvard.edu/pph2/bgi.shtml>) to obtain predictions. For SIFT (vs 4.0.3) the stand alone version was run locally. Again, protein sequences for multiple sequence alignment were retrieved from the NCBI server. The BEST option was used to obtain high-confidence predictions. Only variants which were predicted damaging in both algorithms were considered putatively damaging. In the analyses of relative abundance of variants in the three gene sets, the counts of observed variants were divided by the total length of exonic sequence of the gene to correct for gene length.

To validate results obtained on the 1000G Phase 1 data, we repeated the analyses of individual mutational load in essential genes using the 54 HapMap samples. The analysis of the ratio of non-synonymous and synonymous variants showed a significantly lower ratio in EG when compared to NLG (*P*= 8.36 x 10^-11^) and ALL (*P*= 8.36 x 10^-11^) (Figure S9). When analyzing the gene-length corrected average number of exonic missense variants in EG, NLG and ALL we observed a significantly lower average in the essential genes (*P*= 8.36 x 10^-11^, Figure S10). Note that the identical p-values of *P*= 8.36 x 10^-11^ found in these two analyses are due to the non-overlapping distributions in EG and NLG and ALL respectively. In other words, 8.36 x 10^-11^ is the minimal possible p-value of a paired Wilcoxon test with 54 samples. Analysis of the fraction of loss-of-function (LoF) variants among all exonic missense variants showed a decrease of LoF events in EG relative to NLG (*P*=2.99 x 10^-10^) and ALL (*P*= 8.36 x 10^-11^). Finally, we estimated the number of exonic missense variants, putative damaging variants and loss-of-function (LoF) variants within essential genes in each individual sample. Overall, we find ~1019 (SD 98.6) exonic missense variants within EG in each sample. This includes ~43.4 (SD 6.33) putative damaging variants and ~ 4.88 (SD 2.25) LoF variants (Tables S9 and S10). When stratifying by allele frequency to exlude common variants (frequency >0.05) we observed ~209 (SD 96.7) exonic missense variants in each sample. ~17.4 (SD 6.6) and ~ 2.48 (SD 1.94) variants were putatively damaging or LoF respectively. Further restriction to rare variants (frequency <0.01) yielded ~109.1 (SD 44.6) exonic missense variants. Among these we found 11.2 (SD 4.37) rare putative damaging exonic missense variants and 1.74 (SD 1.54) rare loss-of-function variants in essential genes. These estimates are consistent with the values we obtained from 1000G Phase 1 data (~11 exonic missense variants and ~2 loss-of-function variants).

## Conservation of differential gene expression in essential genes between human and mouse

Large-scale comparative studies of gene expression in mouse and human have shown that there is strong correlation between gene expression patterns of orthologs ([37-39]). To address the question how conserved tissue-specific differential expression in human and mouse within the essential genes is, we obtained data for tissue-specific differential expression for mouse [47] and human [27] from the Gene Expression Atlas (<http://www.ebi.ac.uk/gxa/>). To restrict the analysis to strong differential expression a p-value cutoff of 1e-4 was applied. To explore the degree of conservation in tissue-specific differential expression between human and mouse, we asked the simple question how much more likely it is for a tissue that is differentially expressed in both mouse and human that the direction of differential expression is concordant (both up-regulated or both down-regulated) than discordant (up-regulated in human and down-regulated in mouse or vice versa). Among 21 shared tissues and 1454 essential genes with strong differential expression in at least one tissue, we observed concordant directions of differential expression for 1,292 gene-tissue pairs (human up/mouse up: 958, human down/ mouse down: 334) and discordant directions for 105 gene-tissue pairs (human up/mouse down:68, human down/mouse up:37). Thus, we observe a highly significant enrichment for concordant pairings (Fisher’s test *P*=1.06 x 10^-204^, OR=127.17). This simple analysis illustrates that there is considerable conservation of the direction of differential gene expression in essential genes within corresponding tissues in human and mouse. However, it needs to be stressed that for many genes and tissues differential gene expression is observed only within either human or mouse.

## Additional references:

47. Lattin JE, Schroder K, Su AI, Walker JR, Zhang J, et al. (2008) Expression analysis of G Protein-Coupled Receptors in mouse macrophages. Immunome Res 4: 5.
