# Supplementary material for: From Mouse to Human: Evolutionary Genomics Analysis of Human Orthologs of Essential Genes
Source: PLoS Genet. 2013 May 9;9(5):e1003484. doi: 10.1371/journal.pgen.1003484 (PMC3649967; doi:10.1371/journal.pgen.1003484)
Supplement: Figure S4 — Gene expression profile of SCN3A for mouse (left) and human (right) obtained from the BioGPS website. In both mouse and human there is tissue-specific expression in the brain. (PDF) [file pgen.1003484.s004.pdf]

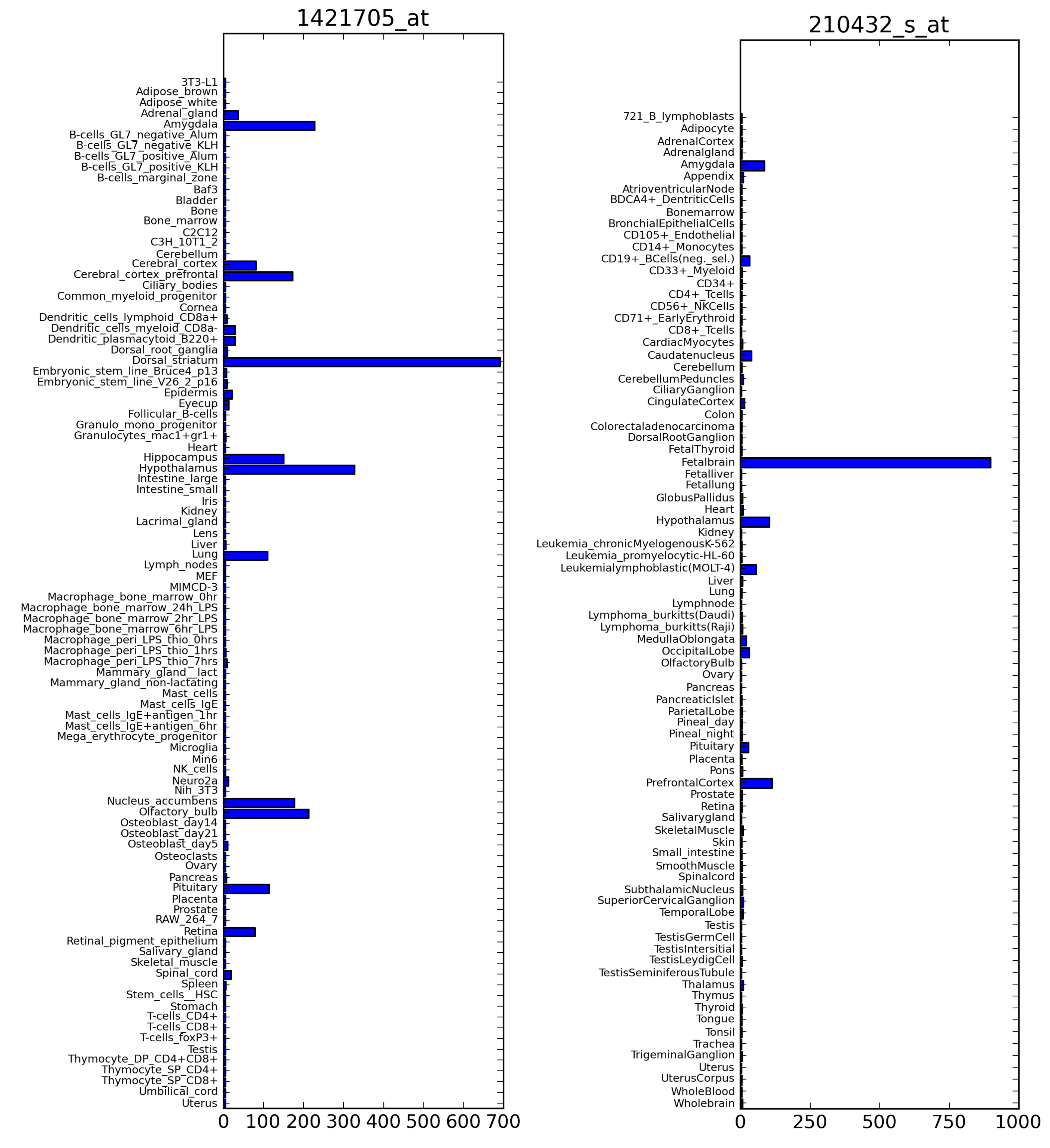
1421705_at
3T3-L1
Adipose_brown
Adipose_white
Adrenal_gland
Amygdala
B-cells_GL7_negative_Alum
B-cells_GL7_negative_KLH
B-cells_GL7_positive_Alum
B-cells_GL7_positive_KLH
B-cells_marginal_zone
Baf3
Bladder
Bone
Bone_marrow
C2C12
C3H_10T1_2
Cerebellum
Cerebral_cortex
Cerebral_cortex_prefrontal
Ciliary_bodies
Common_myeloid_progenitor
Cornea
Dendritic_cells_lymphoid_CD8a+
Dendritic_cells_myeloid_CD8a-
Dendritic_plasmacytoid_B220+
Dorsal_root_ganglia
Dorsal_striatum
Embryonic_stem_line_Bruce4_p13
Embryonic_stem_line_V26_2_p16
Epidermis
Eyecup
Follicular_B-cells
Granulo_mono_progenitor
Granulocytes_mac1+gr1+
Heart
Hippocampus
Hypothalamus
Intestine_large
Intestine_small
Iris
Kidney
Lacrimal_gland
Lens
Liver
Lung
Lymph_nodes
MEF
MIMCD-3
Macrophage_bone_marrow_0hr
Macrophage_bone_marrow_24h_LPS
Macrophage_bone_marrow_2hr_LPS
Macrophage_bone_marrow_6hr_LPS
Macrophage_peri_LPS_thio_0hrs
Macrophage_peri_LPS_thio_1hrs
Macrophage_peri_LPS_thio_7hrs
Mammary_gland__lact
Mammary_gland_non-lactating
Mast_cells
Mast_cells_IgE
Mast_cells_IgE+antigen_1hr
Mast_cells_IgE+antigen_6hr
Mega_erythrocyte_progenitor
Microglia
Min6
NK_cells
Neuro2a
Nih_3T3
Nucleus_accumbens
Olfactory_bulb
Osteoblast_day14
Osteoblast_day21
Osteoblast_day5
Osteoclasts
Ovary
Pancreas
Pituitary
Placenta
Prostate
RAW_264_7
Retina
Retinal_pigment_epithelium
Salivary_gland
Skeletal_muscle
Spinal_cord
Spleen
Stem_cells__HSC
Stomach
T-cells_CD4+
T-cells_CD8+
T-cells_foxP3+
Testis
Thymocyte_DP_CD4+CD8+
Thymocyte_SP_CD4+
Thymocyte_SP_CD8+
Umbilical_cord
Uterus
0
100
200
300
400
500
600
700
210432_s_at
721_B_lymphoblasts
Adipocyte
AdrenalCortex
Adrenalgland
Amygdala
Appendix
AtrioventricularNode
BDCA4+_DentriticCells
Bonemarrow
BronchialEpithelialCells
CD105+_Endothelial
CD14+_Monocytes
CD19+_BCells(neg._sel.)
CD33+_Myeloid
CD34+
CD4+_Tcells
CD56+_NKCells
CD71+_EarlyErythroid
CD8+_Tcells
CardiacMyocytes
Caudatenucleus
Cerebellum
CerebellumPeduncles
CiliaryGanglion
CingulateCortex
Colon
Colorectaladenocarcinoma
DorsalRootGanglion
FetalThyroid
Fetalbrain
Fetalliver
Fetallung
GlobusPallidus
Heart
Hypothalamus
Kidney
Leukemia_chronicMyelogenousK-562
Leukemia_promyelocytic-HL-60
Leukemialymphoblastic(MOLT-4)
Liver
Lung
Lymphnode
Lymphoma_burkitts(Daudi)
Lymphoma_burkitts(Raji)
MedullaOblongata
OccipitalLobe
OlfactoryBulb
Ovary
Pancreas
PancreaticIslet
ParietalLobe
Pineal_day
Pineal_night
Pituitary
Placenta
Pons
PrefrontalCortex
Prostate
Retina
Salivarygland
SkeletalMuscle
Skin
Small_intestine
SmoothMuscle
Spinalcord
SubthalamicNucleus
SuperiorCervicalGanglion
TemporalLobe
Testis
TestisGermCell
TestisIntersitial
TestisLeydigCell
TestisSeminiferousTubule
Thalamus
Thymus
Thyroid
Tongue
Tonsil
Trachea
TrigeminalGanglion
Uterus
UterusCorpus
WholeBlood
Wholebrain
0
250
500
750
1000
